# Extracting Fitness Relationships and Oncogenic Patterns among Driver Genes in Cancer

**DOI:** 10.3390/molecules23010039

**Published:** 2017-12-25

**Authors:** Xindong Zhang, Lin Gao, Songwei Jia

**Affiliations:** 1 School of Computer Science and Technology, Xidian University, Xi’an 710000, China; zxd841@163.com; 2 School of Computer Science, Xi’an Polytechnic University, Xi’an 710000, China; 3 School of Software, Xidian University, Xi’an 710000, China; swjia@xidian.edu.cn

**Keywords:** biological network, driver gene, fitness advantage, oncogenetic pattern

## Abstract

Driver mutation provides fitness advantage to cancer cells, the accumulation of which increases the fitness of cancer cells and accelerates cancer progression. This work seeks to extract patterns accumulated by driver genes (“fitness relationships”) in tumorigenesis. We introduce a network-based method for extracting the fitness relationships of driver genes by modeling the network properties of the “fitness” of cancer cells. Colon adenocarcinoma (COAD) and skin cutaneous malignant melanoma (SKCM) are employed as case studies. Consistent results derived from different background networks suggest the reliability of the identified fitness relationships. Additionally co-occurrence analysis and pathway analysis reveal the functional significance of the fitness relationships with signaling transduction. In addition, a subset of driver genes called the “fitness core” is recognized for each case. Further analyses indicate the functional importance of the fitness core in carcinogenesis, and provide potential therapeutic opportunities in medicinal intervention. Fitness relationships characterize the functional continuity among driver genes in carcinogenesis, and suggest new insights in understanding the oncogenic mechanisms of cancers, as well as providing guiding information for medicinal intervention.

## 1. Introduction

Cancer results from the accumulation of multiple driver mutations in genes [1,2]. Studies have suggested the relation between the acceleration of cancer progression and the accumulation of driver mutations [1,2]. Major efforts have been made in recent years to detect driver genes and recognize mutational combinations among driver genes, mainly focusing on co-occurrence analysis and mutual exclusivity analysis based on the observation that driver mutations in genes in different pathways tend to co-occur, whereas those in the same pathway rarely occur in the same sample (i.e., they are mutually exclusive) [1,3,4,5,6,7,8,9]. Both co-occurrence and mutual exclusivity reveal a non-ordered functional relationship between driver genes.

It has been well accepted that driver mutation provides a fitness advantage to cancer cells, while the accumulation of driver mutations increase the fitness of cancer cells and accelerate cancer progression. Revealing patterns that driver genes have accumulated in carcinogenesis is of greater significance than co-occurrence among driver genes. This is illustrated by the case of APC and TP53 [10]. p53, the production of TP53, is activated by DNA double-strand breaks whose accumulation is led by APC loss, a production of well-known oncogene APC, which implies a real possibility that a driver mutation of APC occurs followed by an aberration of TP53. That is, there is a functional continuity between APC and TP53. TP53 is also called the selective target of APC. The functional continuity between TP53 and APC should be denoted as an order pair <APC, TP53> or a direct edge from APC to TP53, while an unordered pair <APC, TP53> or an undirected edge could be used to characterize the co-occurrence or mutual exclusivity between the two genes. However, few efforts have been made to account for this issue.

The pattern driver mutations accumulated during carcinogenesis are defined as fitness relationship or fitness pattern, characterizing the potential way that driver genes have accumulated to increase the fitness of cancer cells. In the network perspective, fitness relationship can be illustrated as the following process: a driver mutation occurs and destroys the network structure locally, and calls for a new driver mutation to enhance its effect. The network module is employed to characterize the effect of a driver gene on the biological network, and is denoted the driver-induced module (DIM). We argue that the DIM should have the following two properties. (1) DIMs should have the capability to characterize the cancer status, which is the phenotypic indication of fitness advantage; (2) DIMs should be responsible for the differential expression of a number of stressful genes. Based on these two properties of DIMs, we develop a network-based framework to extract fitness relationships among driver genes. The framework firstly detects potential DIMs for each driver gene by a sampling strategy in background networks, and then extracts fitness patterns among driver genes from DIMs. Some interesting results are obtained, which could provide new insights for our understanding of the molecular mechanisms underlying carcinogenesis.

## 2. Materials and Methods

### 2.1. Data Source

All genomic data employed in this work were downloaded from public data portals. Gene expression data for colon adenocarcinoma (COAD) was downloaded from TCGA [11], which contains 51 solid tissue normal samples (NT) and 624 primary solid tumor samples (TP). The gene expression data for skin cutaneous malignant melanoma (SKCM) were downloaded from GEO [12] with the series number GSE15605, which contains 16 normal skin samples and 46 primary melanomas [13]. Gene expression was averaged for duplicated genes and z-score normalized. The somatic mutation data and copy number data for COAD and SKCM were downloaded from TCGA (451 for COAD and 469 for SKCM) and dealt with as described in the available literature [14].

Three types of human gene networks are employed as background networks. The first one was downloaded from the Human Protein Reference Database (HPRD) [15], the second one is a probabilistic functional gene network derived from HumanNet [16], and the third one is a mixed network derived from Wu et al. 2010 [17], and denoted as PPIwu. Additionally, a signaling network [18] was employed for independent validation of functional continuity of driver gene pairs.

### 2.2. Methods

**Figure 1:**
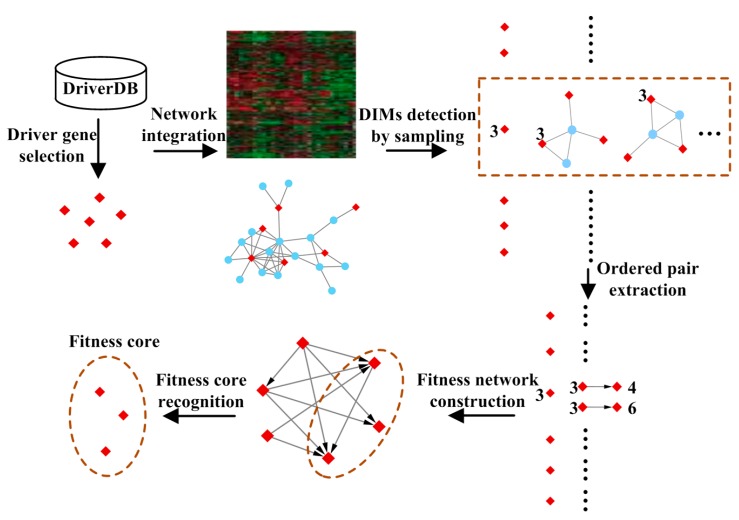
The schematic overview of the proposed framework. Driver genes are collected from DriverDB and a sampling strategy is designed to detect driver-induced modules (DIMs) by integrating gene expression data and network information. Then, fitness relationships among driver genes are extracted from DIMs, which are denoted as weighted ordered pairs. Genes indegree dominated are recognized as the fitness core by network analysis.

The goal of this work is to detect fitness relationships among driver genes in carcinogenesis. A schematic overview of the proposed approach is shown in Figure 1, and described in the following sections.

#### 2.2.1. Driver Gene Selection

Driver genes were collected from DriverDB [19], which provides open access to driver genes for cancers, as identified by well-known algorithms [5,6,7,14,20,21,22,23] from somatic mutations and CNVs, and genes covered by at least two methods were picked out for further study.

#### 2.2.2. Detecting Driver-Induced Modules (DIMs)

A DIM implies a potential local structure for a network affected by a driver gene, and naturally has two properties: the discriminant property of disease status and the enrichment of differentially expressed genes. In light of the functional diversity of a gene, multiple DIMs for a driver gene are under consideration.

We introduce a sampling strategy for generating possible DIMs for each driver gene. The *t*-test score [24] is employed to measure the differentiation ability of a given DIM under particular conditions. Suppose that *M*_k_ is a candidate DIM for a seed *d* with a score *S*_k_ by *k*-th selection, *N*(*M*_k_) denotes network neighbors of *M*_k_, the search randomly adopts an element *u* ∈ *N*(*M*_k_), which yields an increase of *S*_k_ over a threshold, while it terminates if no element is satisfied. For a given gene, this procedure repeats 5000 times, generating 5000 candidate DIMs. Candidate DIMs with enriched differentially expressed genes are picked out and served as DIMs by the Fisher exact test with adjusted *p*-values < 0.05. Differentially expressed genes are generated by the multiple permutation test with adjusted *p*-values < 0.05.

#### 2.2.3. Extracting Ordered Pairs of Driver Genes

Given a driver gene d∈D with corresponding DIMs*^d^*, and an arbitrary driver gene u∈D−{d}, where *D* denotes the universal set of driver genes in the study, an ordered pair d,u is built if *u* is an element of at least one DIM in DIMs*^d^*. The ordered pair d,u can also be explained as a directed edge d→u in graph theory, and implies that *u* is a selective target of *d*. We add a weight *w*_du_ to the ordered pair d,u, where *w*_du_ is the frequency that the ordered pair d,u hits DIMs*^d^*. The triad (d,u, *w*_du_) implies that *u* is a selective target of *d* with a probability *w*_du_. The higher the *w*_du_, the more likely it is for a seed *d* to select *u* as a selective target to enhance its effect on the network.

#### 2.2.4. Construction of the Fitness Network

The fitness network (FN) is constructed by the collection of weighted ordered pairs. Network analysis indicates that more than 90% of shortest-path lengths of fitness networks are less than 3.

#### 2.2.5. Recognition of Fitness Core

The fitness core is defined as a subset of driver genes that is indegree dominated in FN. Genes in the fitness core are served as common selective targets by the majority of driver genes. For a given node *u*, we define the indegree ratio (*IDR*) as follows,

(1) $$IDR\left(u\right)=\frac{Indegree\left(u\right)}{Indegree\left(u\right)+outdegree\left(u\right)}$$

A gene *u* is indegree-dominated if *IDR*(*u*) > *δ*, and *δ* is set to 0.7 in this work.

#### 2.2.6. Absolute Coverage and Relative Coverage

Given a set *M* composed of *t* genes, e.g., *M* = {*g*_1_,*g*_2_L ,*g*_t_}, Γ(*g*) denotes the set of samples with mutations in gene *g*; the coverage of *M* is defined as follows,

(2) $$coverage\left(M\right)=\left|\overset{t}{\underset{i=1}{⋃}}\Gamma \left(g\right)\right|$$

In this work, driver genes are partitioned into two groups: core genes (genes in the fitness core) and non-core genes (genes that exclude core genes). We define relative coverage to measure the identity of tumor samples covered by core genes and non-core genes, while the absolute coverage is defined to indicate the coverage of tumor samples covered by core genes. The formula of the relative coverage is as follows,

(3) $$Relative\;coverage\left(core\right)=\frac{\left|\Gamma \left(core\right)I\;\Gamma \left(non-core\right)\right|}{\left|\Gamma \left(non-core\right)\right|}$$

The absolute coverage is defined as follows,

(4) $$Absolute\;coverage\left(core\right)=\frac{\left|\Gamma \left(core\right)\right|}{\left|\Gamma \left(non-core\right)U\Gamma (core)\right|}$$

#### 2.2.7. Co-Occurrence and Mutual Exclusivity

For two genes *u* and *v*, *U* and *V* are sets of tumor samples with genomic alterations, the significance of co-occurrence and mutual exclusivity of *u* and *v* is determined by Fisher’s exact test, and *p*-values of less than 0.05 are deemed to be of significance.

## 3. Results and Discussion

We apply the framework to COAD and SKCM. The fitness networks constructed are denoted as FN.hp, FN.hu and FN.wu for the background networks HPRD, HumanNet and PPIwu respectively. A fitness network consisting of edges common to FN.hp, FN.hu and FN.wu is denoted as FN.com. A statistics of the results are shown in Table 1.

The fitness cores derived from each case-network combination are listed in Supplementary Table S1, and the fitness cores common to every case is denoted as core3. Finally, seven genes (COL1A2, VCAN, RBL1, SMARCA4, SRC, TP53, and FZD3) are common in three fitness cores in COAD, and six genes (BRAF, CASR, NF1, NRAS, HDAC9, CNTNAP2) are common in SKCM. The IDR variation of core genes with frequency cutoffs are shown in Supplementary Figures S1 and S2.

We introduce a sampling strategy for DIM generation, and the convergence of the sampling strategy is also discussed. Results show that driver genes covered by DIMs are identical when the number of sampling iterations is greater than 1000 (Supplementary Figure S3), which indicates the convergence of the sampling strategy used in the framework.

### 3.1. Validation of Fitness Relationships

#### 3.1.1. Comparison of Fitness Networks

For each case, three fitness networks are derived from different background networks. We compare fitness networks for each case to show whether the results are dependent on the background network. The frequency distributions of the edges in the fitness networks are normalized by the kernel probability distribution with a normal smoothing function. Then, we count the number of edges common to three fitness networks under different weight cutoffs from 0 to 1, with a step 0.005, as well as their corresponding significance. Edges overlapped significantly when the percentage of edges in FN under cutoffs was larger than 30% in all case studies. Additionally, we found that these edges also significantly co-occurred (Supplementary Figure S5). Results indicate that fitness networks generated from different background networks are consistent, which implies the reliability of the fitness networks constructed by our framework.

#### 3.1.2. Cross Validation with Co-Occurrence and Mutual Exclusivity

**Figure 2:**
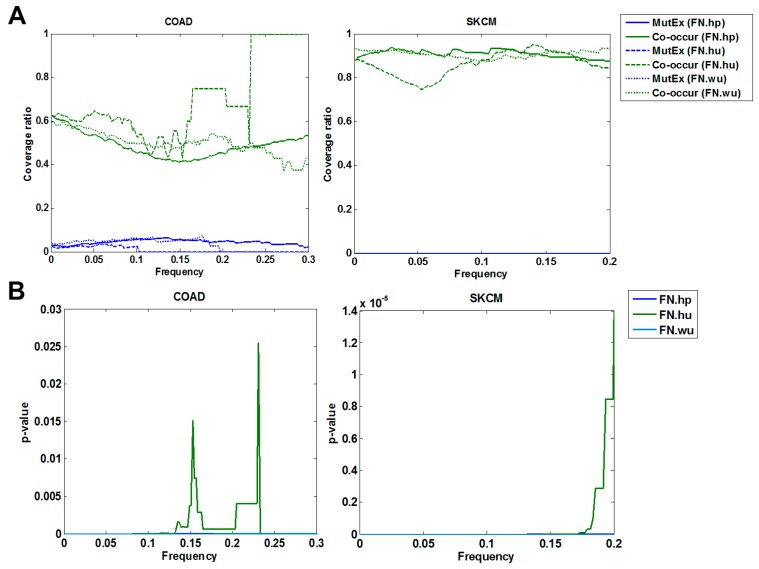
Co-occurrence and mutual exclusivity analysis of fitness relationships. (**A**) The variation of edges of co-occurrence (co-occur) and mutually exclusivity (MutEx) with frequency cutoffs in FNs. (**B**) Significance of co-occurring edges with frequency cutoffs in FNs.

The functional continuity of ordered pairs (edges in FNs) implies potential co-occurrence among driver genes, while edges of mutual exclusivity are unexpected. We validate the co-occurrence of edges in all fitness networks. We calculate the percentage of co-occurred edges in fitness networks with frequency cutoffs of less than 0.3 and 0.2 for COAD and SKCM, respectively, as well as their corresponding significance (Figure 2). The FNs would cover less than 3% of the edges if the frequency cutoffs were higher than 0.3 and 0.2, respectively, for the two case studies. The significance (*p*-value < 0.03) indicates the enrichment of co-occurred edges in FNs.

Edges of mutual exclusivity are under consideration due to their unexpected presence, which is contrary to the functional continuity among driver genes. The percentage of mutually exclusive edges is used to evaluate the false rate introduced by the proposed framework. The false rate is less than 0.06 for all fitness networks in COAD, while no edges of mutual exclusivity exist in the fitness networks in SKCM (Figure 2A).

#### 3.1.3. Cross-Validation with Signaling Network

**Figure 3:**
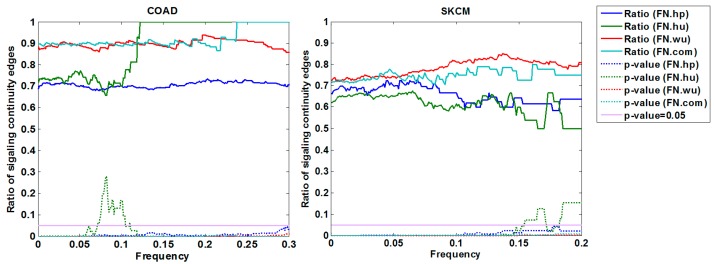
Functional continuity of identified fitness relationships cross-validated by a signaling network. Driver genes in FNs are mapped into an independently collected signaling network. Functional continuity of fitness relationships is maintained by an accessible path from the source nodes to the target ones. The significance *p*-values of fitness relationships with functional continuity are calculated by the Fisher’s exact test.

Additionally, we validate the functional continuity of ordered pairs with the signaling network. Two genes in an ordered pair are of functional continuity if there is an accessible path from the first element to the second one in the signaling network. We examine the functional continuity of all fitness relationships in FNs for each case, and the results showed enriched edges of functional continuity in each fitness network in both cases (Figure 3). In COAD, 53 genes were included in the signaling network, and covered 513 of 669 edges in FN.com, while 460 of them were of functional continuity. In SKCM, 67 genes were included in the signaling network, covering 709 of the 805 edges in FN.com, while 508 of them were of functional continuity. The significance *p*-value was less than 2.2 × 10^−16^ for both cases. Figure 3 shows the fluctuation of the ratio of edges of signaling continuity with frequency cutoffs and corresponding *p*-values.

### 3.2. Analysis of Fitness Core

#### 3.2.1. Fitness Core Are Consistent across Fitness Networks

**Figure 4:**
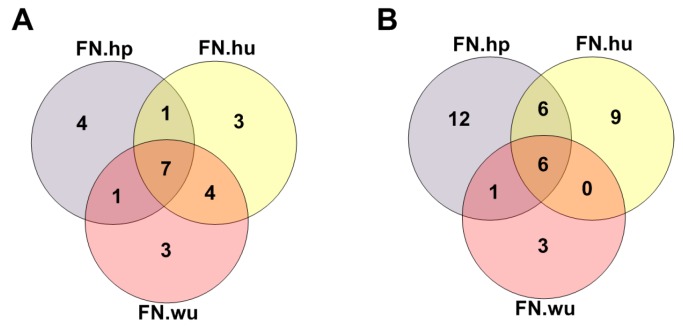
Overlap of fitness cores identified under different background networks (HPRD, Humannet, PPI wu) in COAD (**A**) and SKCM (**B**). Significant overlaps were obtained in both cases with *p*-value < 2.2 × 10^−16^.

For each case, three fitness cores were recognized using the indegree-dominated criterion. A significant overlap (*p*-value < 2.2 × 10^−16^) was obtained among three fitness cores derived from different background networks for each case, indicating that the fitness core was highly consistent across the background networks (Figure 4).

#### 3.2.2. Fitness Core Serves as Common Selective targets of the Majority of Driver Genes

**Figure 5:**
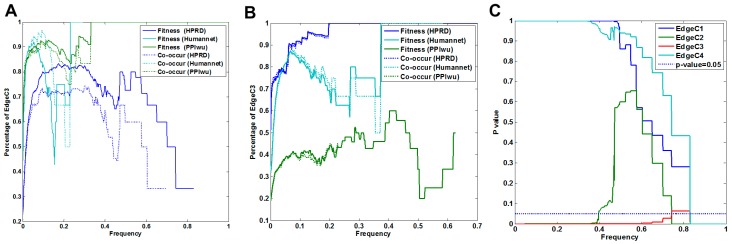

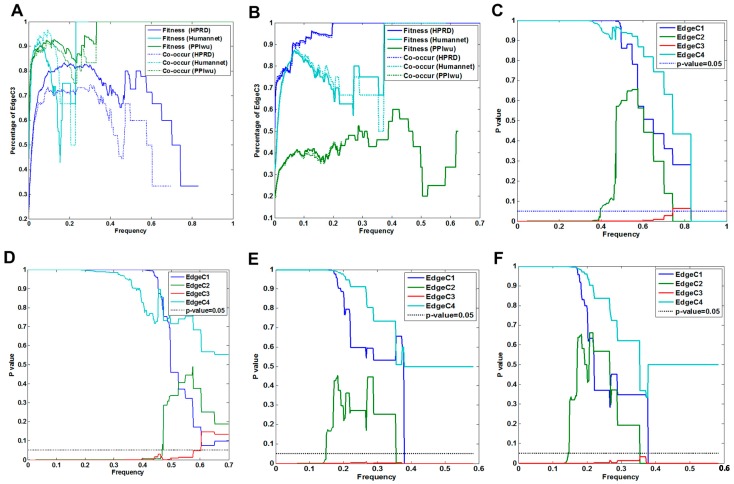
Statistics and significance of edges in each edge class. (**A**,**B**) Variations of fitness edges and co-occurring edges in EdgeC3 under frequency cutoffs in fitness networks in COAD and SKCM (**B**); (**C**,**D**) Significance of fitness edges in each edge class with frequency cutoffs in FN.hp for COAD (**C**) and SKCM (**D**); (**E**,**F**) Significance of co-occurring edges in each edge class with frequency cutoffs in FN.hp for COAD (**E**) and SKCM (**F**). Results show a consistent significance of fitness edges and co-occurring edges in EdgeC3 under different frequency cutoffs.

With the fitness core identified, the nodes in fitness network are separated into two categories. We denote nodes excluding the fitness core as node class I (NodeC1) and those in fitness core are denoted as class II (NodeC2), while edges are correspondingly partitioned into four parts: edges among genes in class I (EdgeC1), edges among genes in class II (EdgeC2), edges starting from genes in class I to genes in class II (EdgeC3), and edges from genes in class II to genes in class I (EdgeC4). We count the number of edges as well as co-occurring edges in each edge class with frequency cutoffs. Results show that edges are non-homogeneously distributed in four edge classes. The majority of both fitness edges and co-occurring edges belong to EdgeC3 across all frequency cutoffs in all fitness networks, in spite of a few exceptions. We also examine the significance of fitness edges and co-occurring edges in four edge classes with frequency cutoffs. Statistics indicate the significance of edges in EdgeC3 (*p*-value < 0.05) across frequencies, despite a few exceptions, while edges in other classes show less significance. The distributions of fitness edges and co-occurring edges with frequency cutoffs are shown in Figure 5A,B, while the variations in significance with frequency cutoffs under HPRD for each case are shown in Figure 5C–F.

The statistics of non-homogeneous distribution of edges in four edge classes indicate that genes in fitness core are more likely to be served as selective targets for the majority of non-core genes in NodeC1, while edges sparsely distributed in EdgeC1 may imply the relative independence of genes in NodeC1 when genomic alterations occur.

#### 3.2.3. Fitness Core Covers the Majority of Tumor Samples by Minor Genes

The functional continuity and enriched co-occurrence between genes in NodeC1 and NodeC2 imply a consistency in the tumor samples covered by genes in the two node classes. We investigate the absolute coverage and the relative coverage of the core genes derived from FNs and FN.com. The absolute coverage and the relative coverage are greater than 0.7 for all fitness cores in COAD and SKCM, and greater than 0.69 for the fitness cores in common. Table 2 shows the relative coverage of the common fitness cores for each case-network combination. Detailed information of the absolute coverage and the relative coverage of fitness cores is provided in Supplementary Tables S2 and S3.

#### 3.2.4. Genes in the Fitness Core Play Key Roles in Carcinogenesis and Therapeutic Intervention

The functional continuity between fitness core and non-core genes also implies the functional importance of the fitness core in carcinogenesis. We validate all genes in the fitness core by literation study. In total, 24 and 36 genes are included in the fitness cores for COAD and SKCM, respectively. In COAD, 16 of the 24 genes have been reported to be essential in colonic carcinogenesis, while 13 of them are common to at least two fitness cores, and 12 genes are reported in the literature, including some well-studied genes, such as BMP7 [25,26], RBL1 [27,28,29], SMARCA4 [30,31,32], SRC [33,34,35], and TP53 [36,37,38]. In SKCM, 19 of the 36 genes have been reported to play key roles in malignant melanoma carcinogenesis and metastasis, while 12 genes are common to at least two fitness cores, of which 7 genes have been reported in the literature, including some well-studied genes, including BRAF [39,40,41], MDC1 [42,43], NF1 [44,45,46,47], NRAS [48,49,50,51], and PTEN [52,53,54]. Furthermore, 10 genes and 7 genes had been reported as biomarkers or potential therapeutic targets for each case, respectively. Interestingly, for COAD, all 7 genes in the core had been literature-reported to be colonic-carcinogenesis-important, and 5 genes (RBL1, SRC, SMARCA4, TP53 and VCAN) had been reported as gene biomarkers or potential therapeutic targets [27,34,38,55,56], including drug targets associated by database query in DGIdb [57]. In SKCM, all 6 genes in the core had been literature-reported as carcinogenesis-important; of these, 5 genes (BRAF, CASR, NF1, NRAS and HDAC9) had been reported as gene biomarkers or potential therapeutic targets [40,50,57] (Supplementary Tables S4 and S5).

### 3.3. Pattern Analysis

**Figure 6:**
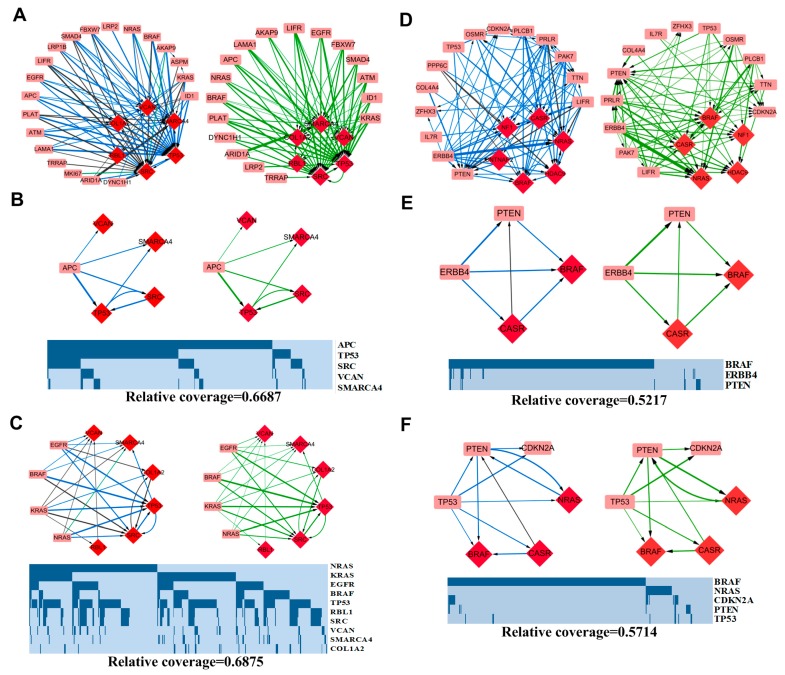
Fitness networks of the top mutated genes in FN.com (red diamond), edges of functional continuity with mean frequencies higher than 0.01, as well as their mutational profiles. Edges of functional continuity, co-occurrence and mutual exclusivity are colored green, dark blue, and light blue, respectively. A thicker edge corresponds to a higher mean frequency. (**A**) Fitness network for COAD. (**B**) APC-related patterns for COAD; (**C**) MAPK pathway-related patterns for COAD; (**D**) Fitness network for SKCM; (**E**) TP53-related patterns for SKCM; (**F**) ERBB4-related patterns for SKCM.

We analyze the fitness relationships among driver genes with higher mutation rates in each case. Specifically, the top 20 mutated genes and top 14 mutated genes with corresponding fitness cores are under consideration for COAD and SKCM. Figure 6 shows the network structure common to all fitness networks for each case.

#### 3.3.1. APC-Related Patterns in COAD

As a “gatekeeping” gene, APC, whose gene product acts as an antagonist of the Wnt signaling pathway, initiates colorectal neoplasia and is frequently mutated in patients with colon cancer [58,59]; mutations that occur in 75.55% (346/458) of COAD samples. APC is a gene with higher outdegree but lower indegree in all three fitness networks (Supplementary Figure S1). In total, APC selects 56 driver genes as its selective targets in all fitness networks in COAD, while 20 of them are common to three fitness networks (Figure 6A), and six genes (TP53, RAE1, SRC, VCAN, SMARCA4, MAPRE1) have frequencies higher than 0.01, of which 4 genes (TP53, SRC, VCAN, SMARCA4) are common to three fitness cores (Figure 6B). The functional continuity of the ordered pair <APC, TP53> has been reported in a previous study [10].

#### 3.3.2. MAPK Signaling Pathway-Related Patterns in COAD

The MAPK signaling pathway is a key pathway in carcinogenesis, and four genes—EGFR, KRAS, NRAS and BRAF—are frequently mutated in COAD. In FN.com, 17 genes in total are served as selective targets for these four genes, with 12 common genes, including 5 core genes (Figure 6C), which implies similar functions for the four genes in carcinogenesis, and is consistent with the assumption of mutual exclusivity.

In the selective targets of the four genes, TP53 obtains the highest mean frequencies, and co-occurs with all four genes. The selection of EGFR, KRAS, NRAS and BRAF to TP53 implies the necessity of the functional changes of TP53 when genomic alterations occur in EGFR, KRAS, NRAS and BRAF in colonic carcinogenesis, which is consistent with the cooperation of MAPK signaling and TP53 discussed in publications [60,61].

Furthermore, 10 genes serve as selective targets for TP53 in COAD, including BMP7, COL1A2, MAPRE1, PFDN4, RAE1, RBL1, SMARCA4, SRC, SUPT5H and VCAN. All of these 10 genes are fitness core genes, and 2 fitness relationships, <TP53, SMARCA4> and <TP53, SRC> exhibit frequencies greater than 0.01 in all FNs.

Contrary patterns are observed in SKCM (Figure 6D). As the most mutated gene in SKCM, BRAF widely participates in the melanomagenesis. In conjunction with NRAS, a mutually exclusive partner in the RAS/MAPK cascade, BRAF covers nearly 60% of samples with melanoma. In FN.com, BRAF and NRAS serve as the selective targets of 42 genes in all 67 genes, and co-occur with 65 genes (see details in Supplementary Table S6), while in total, 7 genes serve as selective targets for BRAF and NRAS (see details in Supplementary Table S7), of which 6 genes, BCLAF1, FLT1, GRIN2A, HDAC9, MDC1 and PTEN, are recognized as fitness core genes. All of this implies the convergence role of the MAPK pathway in melanomagenesis, and the therapeutic opportunities represented by the RAS/MAPK cascade [62,63,64].

#### 3.3.3. ERBB4 Related Patterns in SKCM

**Figure 7:**
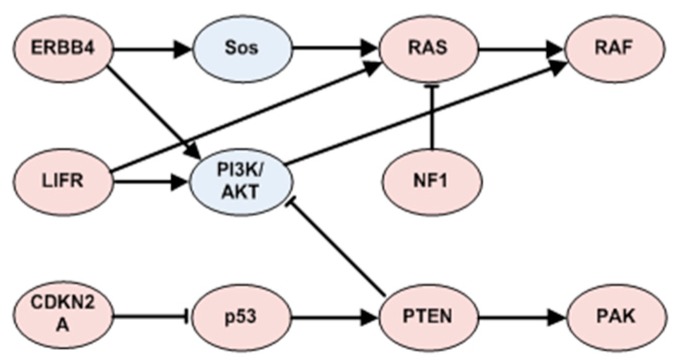
A concise exhibition of functional continuity among top mutated driver genes for SKCM.

Among the top 20 mutated driver genes in FN.com in SKCM, ERBB4 exhibits the highest outdegree and the lowest indegree (Supplementary Figure S2), which implies that lots of genes would be functionally altered when genomic alterations occur in ERBB4. There is also significant co-occurrence between ERBB4 and all 15 selective targets in the top 20 mutated genes observed, all of which implies a dominant role for ERBB4 in melanomagenesis as well as the therapeutic opportunity represented by ERBB4 [65,66,67]. Figure 6E shows ERBB4-related patterns with frequencies larger than 0.01. A concise depiction of functional relationships is shown in Figure 7, which is extracted from KEGG [68].

#### 3.3.4. TP53 Related Patterns in SKCM

Contrary to COAD, TP53 is a non-core gene in SKCM. It employs five genes (BRAF, CASR, CDKN2A, NRAS, and PTEN) as its selective targets in the top 20 mutated genes in FN.com, and co-occurs with them (Figure 6F).

CDKN2A has been reported to be highly correlated with melanoma [69,70]. Two of its encoded proteins are p14ARF and p16INK4a. By binding to MDM2, p14ARF inhibits MDM2-induced degradation of p53, and enhances p53-dependent transactivation and apoptosis, while p16INK4a acts as a negative regulator for the proliferation of normal cells by interacting strongly with CDK4 and CDK6, which is a critical downstream of p53 in G1/S progression [68,71]. The high correlations of p14ARF, p16INK4a and p53 imply the necessity of a functional switch of p14ARF, p16INK4a to p53 loss in melanomagenesis. Another selective target of TP53 is PTEN, which is regulated by p53, and plays key roles in the inhibition of the IGF-1/mTOR pathway. TP53 also serves BRAF and NRAS as selective targets to enhance the effect of the function loss of its gene product, p53, which would be characterized by the p53-TPEN-AKT-BRAF cascade in the PI3K-AKT pathway.

## 4. Conclusions

Combinatorial patterns among driver genes can contribute to cancer intervention, prognosis and a good understanding of the molecular mechanisms underlying cancer progression. Fitness relationship of driver genes characterizes the way driver genes accumulated to increasing cancer progression. However, major efforts in this field are mainly focused on co-occurrence and mutual exclusivity among driver genes, which are unable to characterize the selective tendencies of driver genes adequately. In this work, we introduced a systematic framework for the first time to explore the fitness relationships of driver genes.

Driver mutation provides fitness advantages to cancer cells, and the accumulation of which leads to detrimental changes to cell status. From a network perspective, it induces numerous modules with discriminative capacity for cancer status, and induces the enrichment of differentially expressed genes. Based on this assumption, fitness relationships are detected by the designed framework and validated by network analysis. The results derived from multiple validations indicate that fitness relationships are more conducive to cancer intervention, drug target design and prognosis, and provide a new insights for developing a good understanding of the mechanisms of cancer. Furthermore, the overwhelming majority of edges from non-core genes to genes in the fitness core exhibit oncogenetic patterns in carcinogenesis.

In conclusion, the designed framework is efficiently captures the fitness relationships and oncogenetic patterns behind cancer progression. Case studies in COAD and SKCM also suggest widespread implications of the framework for other types of cancers.

## Figures and Tables

**Table 1:** Statistics of results obtained in case studies.

Cancer Type (Background Network)	Number of Driver Genes (Valid/All)	Fitness Network (Node/Edge)	Number of Genes in Fitness Core
COAD (HPRD)	102/120	98/4021	13
COAD (Huannet)	117/120	100/2705	15
COAD (PPIwu)	95/120	62/922	15
SKCM (HPRD)	129/197	98/1798	25
SKCM (Humannet)	147/197	140/6912	21
SKCM (PPIwu)	117/197	92/2512	10

**Table 2:** Relative coverage and absolute coverage of samples covered by fitness cores in common for COAD and SKCM.

FN-Cancer	Number of Core Genes in Common	The Relative Coverage	The Absolute Coverage
FN.hp-COAD	7	(440, 307, 0.6977)	(440, 307, 0.6977)
FN.hu-COAD	7	(439, 307, 0.6993)	(439, 307, 0.6993)
FN.wu-COAD	7	(439, 307, 0.6993)	(439, 307, 0.6993)
FN.hp-SKCM	6	(167, 123, 0.7365)	(350, 298, 0.8514)
FN.hu-SKCM	6	(195, 144, 0.7385)	(357, 298, 0.8347)
FN.wu-SKCM	6	(138, 99, 0.7174)	(348, 298, 0.8563)

## Supplementary Materials

The following are available online.

Figure S1: Variation of indegree ratio with frequency cutoffs from 0 to 0.2 with a step 0.002 for fitness core in COAD.

Figure S2: Variation of indegree ratio with frequency cutoffs from 0 to 0.2 with a step 0.002 for fitness core in SKCM.

Figure S3: The convergence of sampling strategy.

Figure S4: Frequency distribution of fitness edges and co-occurred edges.

Figure S5: Percentage of the number of fitness and co-occurred edges with frequency cutoffs and corresponding significance.

Figure S6: Overlap of fitness cores identified under different fitness networks in COAD and SKCM.

Figure S7: A concise exhibition of functional relationships among top mutated driver genes for SKCM.

Table S1: Fitness core for each case-background-network combination.

Table S2: Absolute coverage of fitness cores.

Table S3: Relative coverage of fitness cores

Table S4: Functional and therapeutic implications of core genes in COAD.

Table S5: Functional and therapeutic implications of core genes in SKCM.

Table S6: Fitness relationships common in FNs for COAD with corresponding frequency.
