# Supplementary material for: Extracting Fitness Relationships and Oncogenic Patterns among Driver Genes in Cancer
Source: Molecules. 2017 Dec 25;23(1):39. doi: 10.3390/molecules23010039 (PMC5943933; doi:10.3390/molecules23010039)
Supplement: Supplementary file 1 [file molecules-23-00039-s001.pdf]

Supplementary file

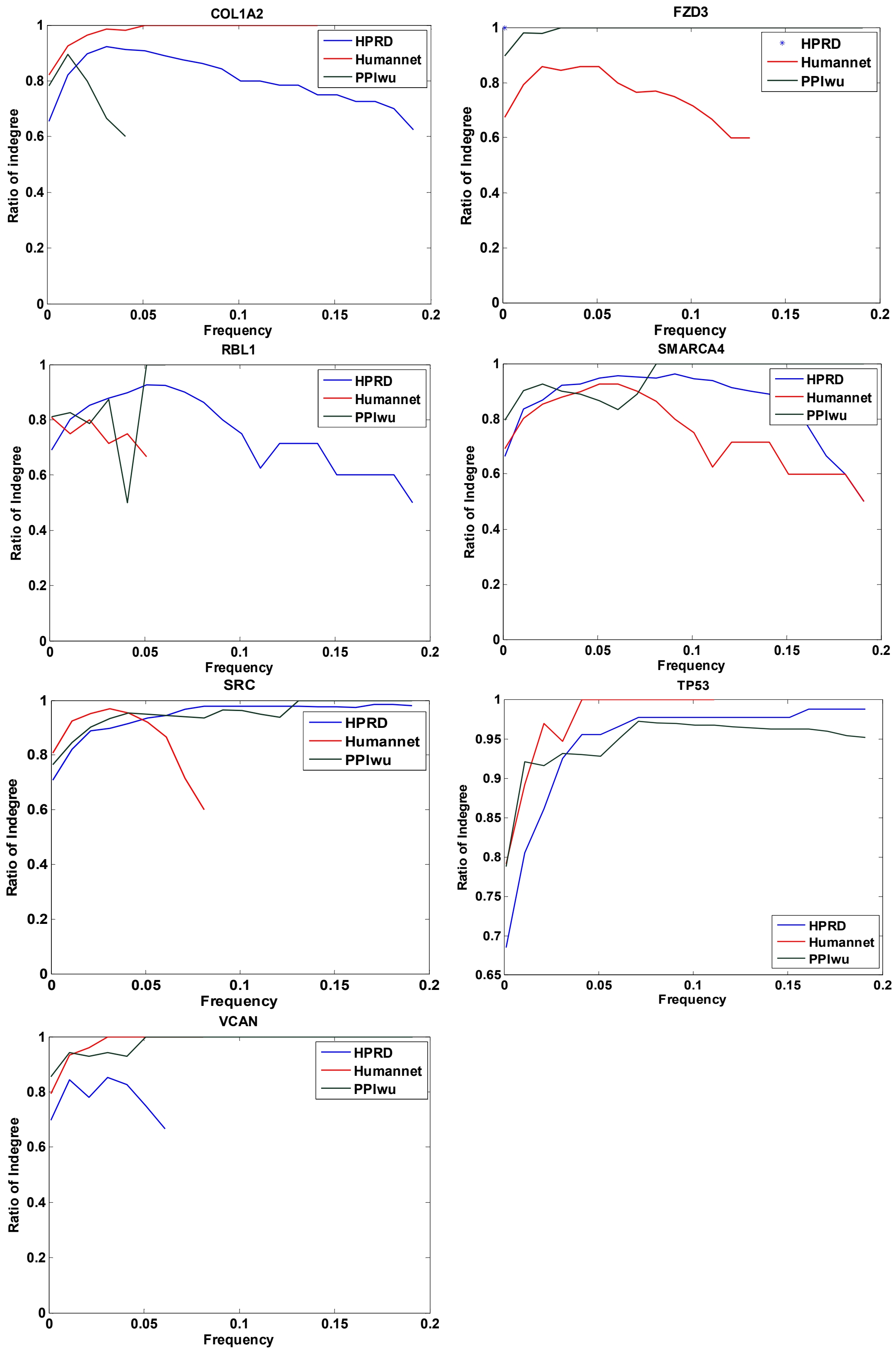

Figure S1. Variation of indegree ratio with frequency cutoffs from 0 to 0.2 with a step 0.002 for driver genes common by three fitness cores in COAD. The indegree ratio is set to NULL if total degree of this genes is less than 5.

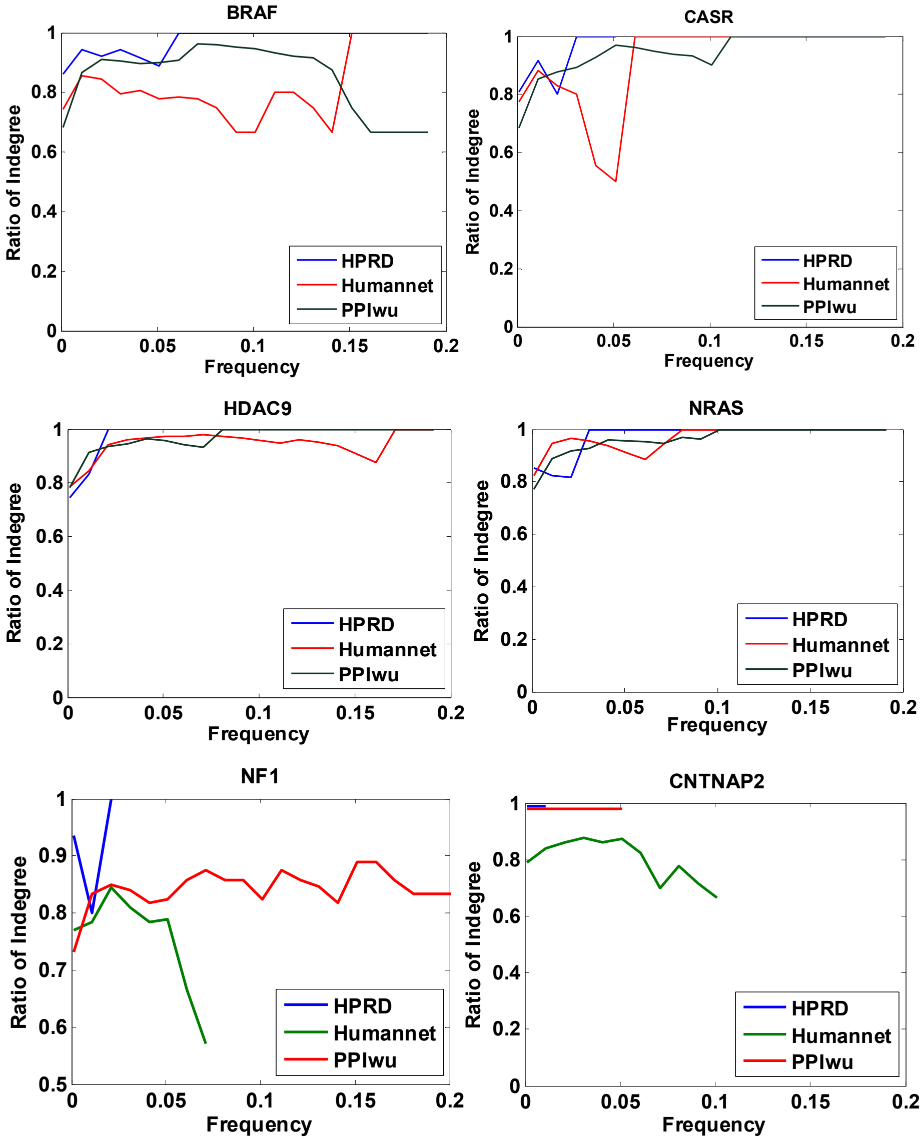

Figure S2. Variation of indegree ratio with frequency cutoffs from 0 to 0.2 with a step 0.002 for driver genes common by three fitness cores in primary melanoma. The indegree ratio is set to NULL if total degree of this genes is less than 5.

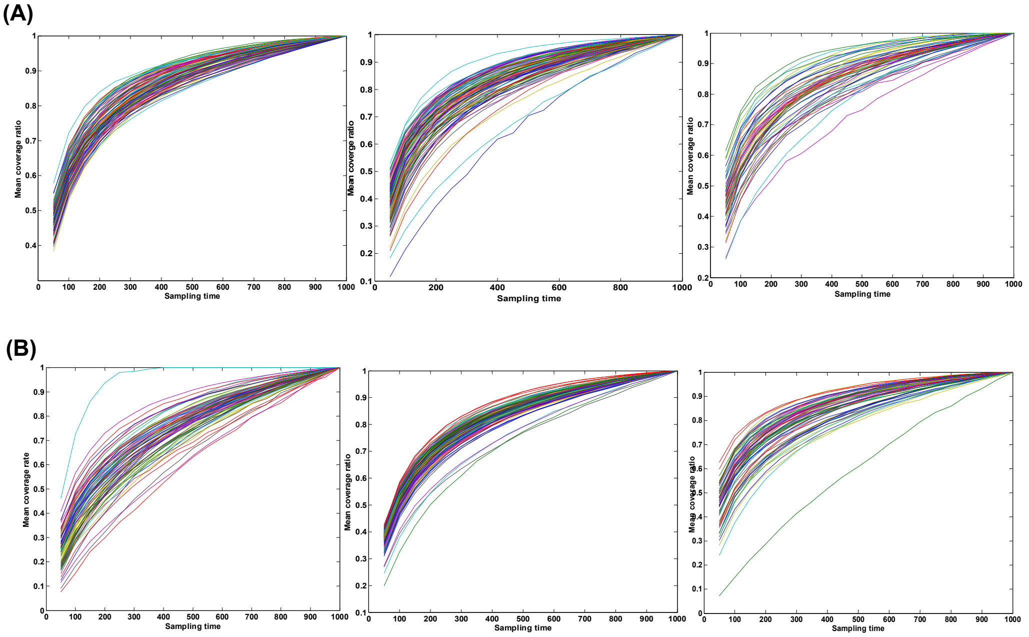

Figure S3. The convergence of sampling strategy in combinations of cases and background networks. (A) Mean coverage of driver genes in COAD with sampling times under HPRD (left), Humannet (middle) and PPIwu (right); (B) Mean coverage of driver genes in primary with sampling times under HPRD (left), Humannet (middle) and PPIwu (right). For a given driver gene and a case-background network combination, a universal set of driver genes covered by DIMs are generated by a 5000 times sampling procedure and DIM filtering procedure, and also subsets of DIM covered driver genes are generate by the same procedures with sampling time from 50 to 5000. Mean coverage is calculated for each sampling time by 100 repeats. The mean coverage is larger than 0.98 for almost all driver genes in each combination when sampling time get up to 1000, which implies the convergence of the sampling strategy employed in the flowchart.

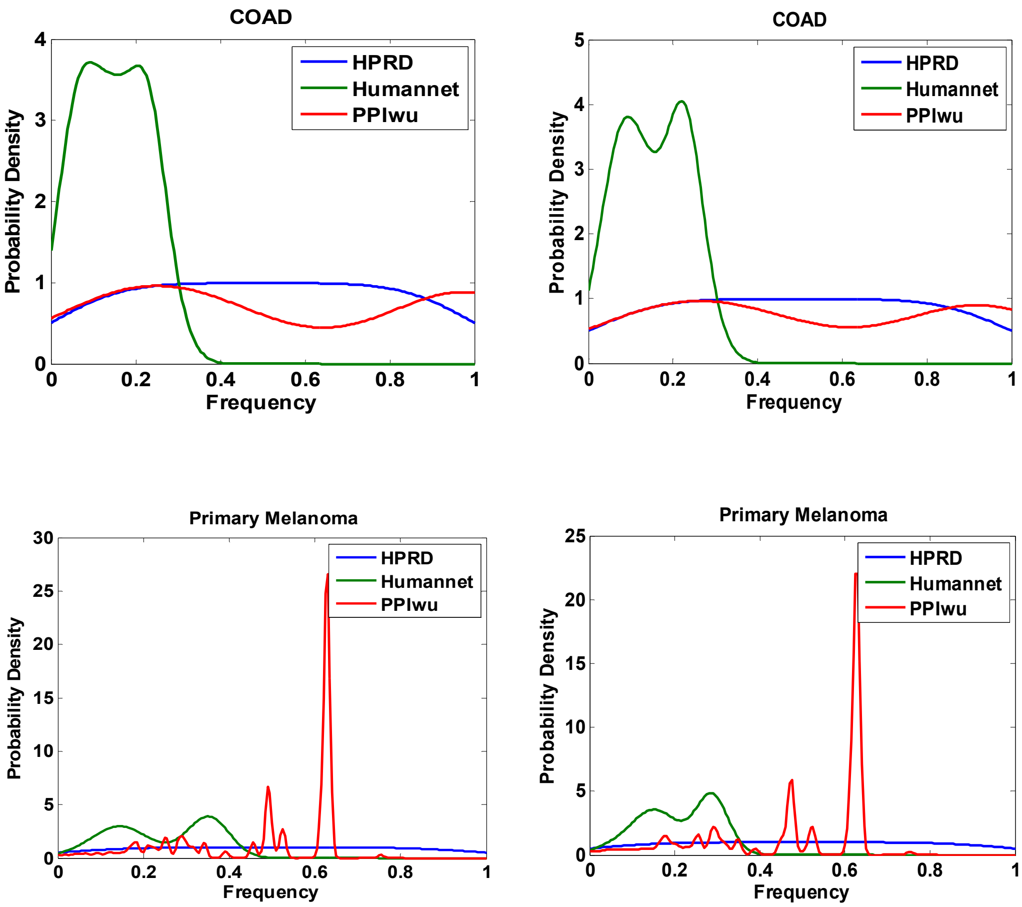

Figure S4. Frequency distribution of fitness (left) and co-occurred (right) edges modeled by the kernel probability distribution with normal smoothing function. Results show significant differences of frequency distribution in different fitness networks.

A

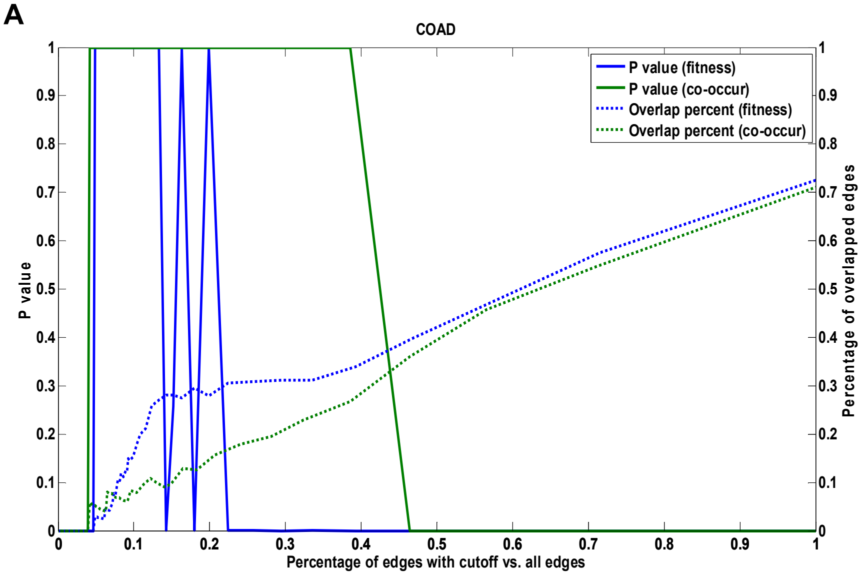

B

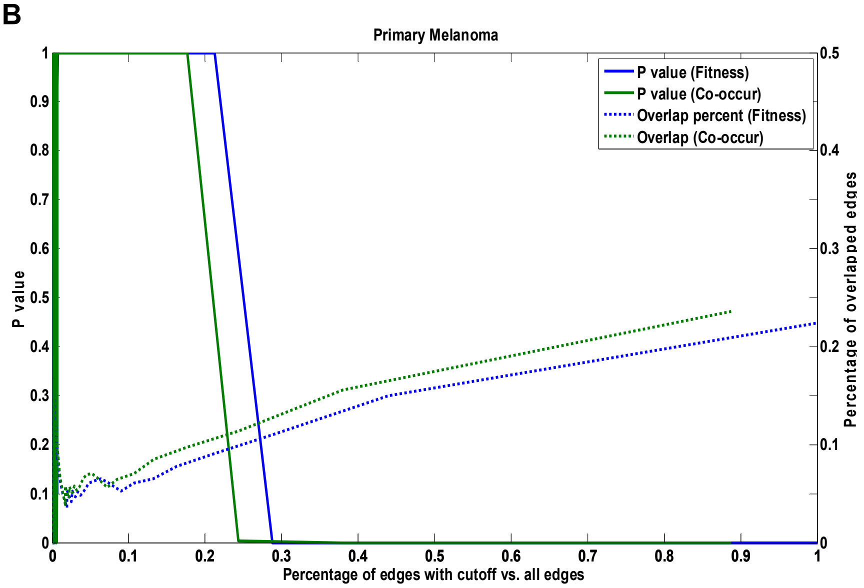

Figure S5. Percentage of the number of fitness and co-occurred edges common by fitness networks with frequency cutoffs and corresponding significance. Results indicate that consistent significances of fitness edges are obtained when percentage of edges in fitness networks larger than 0.23 and 0.28 for COAD and primary melanoma respectively, as well as 0.47 and 0.25 for co-occurred edges for COAD and primary melanoma respectively.

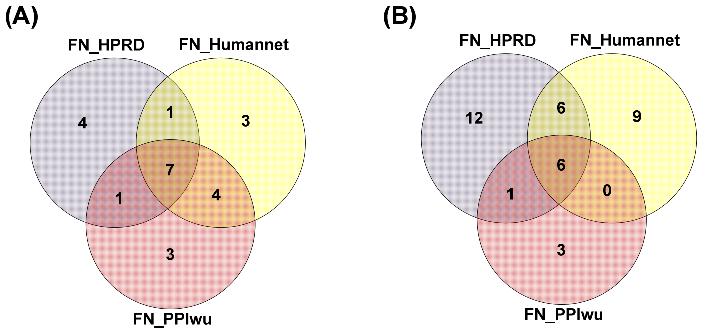

Figure S6. Overlap of fitness cores identified under different background networks (HPRD, Humannet, PPIwu) in COAD (Figure A) and primary melanoma (Figure B). Significant overlaps are obtained in both of cases with p-value < 2.2E-16.

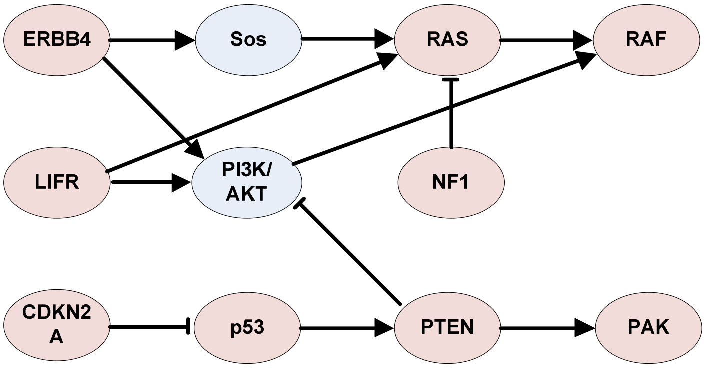

Figure S7. A concise exhibition of functional relationships among top mutated driver genes for SKCM.

Table S1. Fitness core obtained by combinations of cases and background networks

**cancer type**	**Fitness core in FNs**	**Fitness core in common**
COAD (HPRD)	BMP7, COL1A2, VCAN, LRP2, RBL1, SMARCA4, SRC, TP53, USH2A, FZD3, TOMM34, NCOA6, PCDH17	
COAD (Huannet)	BMP7, COL1A2, COL5A1, VCAN, DNAH5, PFDN4, RBL1, SMARCA4, SRC, TP53, UBE2V1, FZD3, RAE1, TCFL5, MXRA5	COL1A2, VCAN, RBL1, SMARCA4, SRC, TP53, FZD3
COAD (PPIwu)	COL1A2, COL5A1, VCAN, PFDN4, RBL1, SMARCA4, SRC, TP53, UBE2V1, FZD3, RAE1, TOMM34, MAPRE1, NEURL2, ASPM	
SKCM (HPRD)	ACTN2, ZFHX3, BRAF, CASR, CDKN2A, CHGB, LYST, COL4A1, COL5A2, DSCAM, FLT1, GRM8, KDR, KEL, NF1, NID1, NRAS, ATXN1, SELE, CD163,	BRAF, CASR, NF1, NRAS, HDAC9, CNTNAP2

	MDC1, HDAC9, ADAM28, CNTNAP2, KCNQ5
SKCM (Humannet)	ACTN2, ANK1, APOB, ZFHX3, BRAF, CASR, LYST, DSCAM, BPTF, GRIN2A, NF1, NRAS, PPP6C, PTEN, ATXN1, TTN, KMT2D, MDC1, HDAC9, CNTNAP2, CHD6
SKCM (PPIwu)	BRAF, CASR, CDKN2A, CLCN1, NF1, NRAS, HDAC9, BCLAF1, CNTNAP2, PCDH18

Table S2 Absolute coverage of fitness cores

FN	num.all	coverage.all	num.core	coverage.core	num.core3	coverage.core3
COAD.hp	16	(440,271,0.8432)	13	(440,342,0.7773)	7	(440,307,0.6977)
COAD.hu	25	(439,404,0.9203)	15	(439,337,0.7677)	7	(439,307,0.6993)
COAD.wu	17	(439,334,0.7608)	15	(439,319,0.7267)	7	(439,307,0.6993)
SKCM.hp	27	(350,315,0.9000)	25	(350,310,0.8857)	6	(350,298,0.8514)
SKCM.hu	28	(357,334,0.9076)	21	(357,323,0.9048)	6	(357,298,0.8347)
SKCM.wu	17	(348,316,0.9080)	10	(348,304,0.8736)	6	(348,298,0.8563)

Table S3 Relative coverage of fitness cores

FN	num.all	coverage.all	num.core	coverage.core	num.core3	coverage.core3
COAD.hp	16	(440,371,0.8432)	13	(440,342,0.7773)	7	(440,307,0.6977)
COAD.hu	25	(439,404,0.9203)	15	(439,337,0.7677)	7	(439,307,0.6993)
COAD.wu	17	(438,333,0.7603)	15	(439,319,0.7267)	7	(439,307,0.6993)
SKCM.hp	27	(150,115,0.7667)	25	(156,116,0.7436)	6	(167,123,0.7365)
SKCM.hu	28	(169,136,0.8047)	21	(172,138,0.8023)	6	(195,144,0.7385)
SKCM.wu	17	(128,99,0.7734)	10	(143,102,0.7133)	6	(138,99,0.7174)

* FN: Fitness network. hp: HPRD; hu: Humannet; wu: PPIwu. all: Indegree-dominate genes derived on fitness networks with frequency cutoff 0.001. num.xx: the number of xx. coverage.core: the coverage of core. core3: fitness core common by three fitness networks.

Table S4 Functional and therapeutic implications of genes common by fitness cores in COAD

gene symbol	Functional implications in literature	Biomarker/Target in literature	Association in DGIdb

COL1A2	PMID: 22351925; PMID: 2998604; PMID: 8280471		
VCAN	PMID: 24951259; PMID: 22711178	Biomarker/Target	
RBL1	PMID: 19568282; PMID: 17979151; PMID: 11237530; PMID: 8817077	Target	
SMARCA4	PMID: 18437052; PMID: 25169151; PMID: 19234488		DGIdb
SRC	PMID: 9444956; PMID: 9018114; PMID: 18477987;		DGIdb
TP53	PMID: 9622060; PMID: 9495355; PMID: 15131045;	Biomarker/Target	DGIdb
FZD3	PMID: 21181886; PMID: 26809274;		

Table S5 Functional and therapeutic implications of genes common by fitness cores in SKCM

**Gene**	**Function implication by literature**	**biomarker/Target**	**Association in DGIdb**
BRAF	PMID: 21343559; PMID: 21639808; PMID: 20818844; PMID: 20823850; PMID: 14679157	biomarker/Target	DGIdb
CASR	PMID: 19237714; PMID: 23267858		DGIdb
NF1	PMID: 24576830; PMID: 8516298; PMID: 15057048; PMID: 13424854; PMID: 25893129; PMID: 7917998; PMID: 23171796		DGIdb
NRAS	PMID: 15009714; PMID: 14695152; PMID: 22180178; PMID: 22614978; PMID: 22453013; PMID: 17119447	Biomarker/Target	DGIdb
HDAC9	PMID: 22959022;		DGIdb
CNTNAP2			

Table S6 Fitness relationships common in FNs for COAD with corresponding frequency

Gene 1	Entrez ID	Gene 2	Entrez ID	Frequency_HPRD	Frequency_Humannet	Frequency_PPIwu

ACTN4	81	ASPM	259266	0.020661157	0.003525264	0.007423118
ACTN4	81	COL1A2	1278	0.05785124	0.03760282	0.069989396
ACTN4	81	COL5A1	1289	0.001033058	0.019976498	0.102863203
ACTN4	81	FZD3	7976	0.001033058	0.014101058	0.064687169
ACTN4	81	NRAS	4893	0.007231405	0.001175088	0.001060445
ACTN4	81	PFDN4	5203	0.016528926	0.008225617	0.004241782
ACTN4	81	RAE1	8480	0.004132231	0.016451234	0.001060445
ACTN4	81	RBL1	5933	0.05268595	0.001175088	0.011664899
ACTN4	81	SMARCA4	6597	0.074380165	0.017626322	0.009544008
ACTN4	81	SRC	6714	0.190082645	0.023501763	0.113467656
ACTN4	81	SUPT5H	6829	0.004132231	0.008225617	0.001060445
ACTN4	81	TOMM34	10953	0.032024793	0.009400705	0.004241782
ACTN4	81	TP53	7157	0.356404959	0.010575793	0.305408271
ACTN4	81	VCAN	1462	0.04338843	0.025851939	0.001060445
ACTN4	81	WWOX	51741	0.020661157	0.001175088	0.005302227
AKAP9	10142	ASPM	259266	0.031982942	0.013017751	0.010638298
AKAP9	10142	BMP7	655	0.029850746	0.026035503	0.003191489
AKAP9	10142	BMPR2	659	0.007462687	0.009467456	0.00212766
AKAP9	10142	CEP250	11190	0.024520256	0.009467456	0.330851064
AKAP9	10142	COL1A2	1278	0.071428571	0.068639053	0.007446809
AKAP9	10142	MAPRE1	22919	0.001066098	0.018934911	0.172340426
AKAP9	10142	NEURL2	140825	0.068230277	0.002366864	0.008510638
AKAP9	10142	NRAS	4893	0.011727079	0.00591716	0.004255319
AKAP9	10142	PCDH17	27253	0.026652452	0.007100592	0.029787234
AKAP9	10142	PFDN4	5203	0.018123667	0.020118343	0.020212766
AKAP9	10142	RAE1	8480	0.007462687	0.030769231	0.113829787

AKAP9	10142	RBL1	5933	0.054371002	0.002366864	0.004255319
AKAP9	10142	SMARCA4	6597	0.10021322	0.134911243	0.015957447
AKAP9	10142	SRC	6714	0.16098081	0.035502959	0.05
AKAP9	10142	SUPT5H	6829	0.017057569	0.021301775	0.00212766
AKAP9	10142	TOMM34	10953	0.026652452	0.013017751	0.121276596
AKAP9	10142	TP53	7157	0.377398721	0.020118343	0.140425532
AKAP9	10142	UBE2V1	7335	0.009594883	0.022485207	0.058510638
AKAP9	10142	VCAN	1462	0.05010661	0.027218935	0.024468085
AKAP9	10142	WWOX	51741	0.034115139	0.009467456	0.00106383
APC	324	ASPM	259266	0.027203482	0.006329114	0.006825939
APC	324	BMP7	655	0.01523395	0.039240506	0.002275313
APC	324	CASP8	841	0.052230686	0.002531646	0.001137656
APC	324	COL1A2	1278	0.04570185	0.067088608	0.004550626
APC	324	MAPRE1	22919	0.368879217	0.098734177	0.023890785
APC	324	NCOA6	23054	0.025027203	0.002531646	0.005688282
APC	324	NEURL2	140825	0.079434168	0.002531646	0.003412969
APC	324	NRAS	4893	0.004352557	0.003797468	0.004550626
APC	324	PCDH17	27253	0.026115343	0.003797468	0.030716724
APC	324	PFDN4	5203	0.140369967	0.036708861	0.009101251
APC	324	RAE1	8480	0.117519042	0.036708861	0.097838453
APC	324	RBL1	5933	0.070729053	0.006329114	0.032992036
APC	324	SMARCA4	6597	0.118607182	0.034177215	0.045506257
APC	324	SRC	6714	0.215451578	0.06835443	0.071672355
APC	324	SUPT5H	6829	0.004352557	0.012658228	0.005688282
APC	324	TOMM34	10953	0.028291621	0.016455696	0.006825939
APC	324	TP53	7157	0.386289445	0.051898734	0.203640501

APC	324	TRRAP	8295	0.008705114	0.002531646	0.004550626
APC	324	UBE2V1	7335	0.002176279	0.046835443	0.034129693
APC	324	VCAN	1462	0.017410229	0.034177215	0.052332196
ARID1A	8289	ASPM	259266	0.010214505	0.002439024	0.003496503
ARID1A	8289	BMP7	655	0.026557712	0.03902439	0.001165501
ARID1A	8289	COL1A2	1278	0.035750766	0.048780488	0.008158508
ARID1A	8289	COL5A1	1289	0.00102145	0.02195122	0.01048951
ARID1A	8289	FZD3	7976	0.002042901	0.031707317	0.201631702
ARID1A	8289	MAPRE1	22919	0.007150153	0.01097561	0.001165501
ARID1A	8289	NEURL2	140825	0.048008172	0.001219512	0.003496503
ARID1A	8289	NRAS	4893	0.004085802	0.006097561	0.002331002
ARID1A	8289	PCDH17	27253	0.032686415	0.001219512	0.031468531
ARID1A	8289	PFDN4	5203	0.025536261	0.13902439	0.011655012
ARID1A	8289	RAE1	8480	0.006128703	0.020731707	0.006993007
ARID1A	8289	RBL1	5933	0.074565884	0.006097561	0.004662005
ARID1A	8289	SMARCA4	6597	0.830439224	0.069512195	0.145687646
ARID1A	8289	SRC	6714	0.219611849	0.042682927	0.107226107
ARID1A	8289	SUPT5H	6829	0.010214505	0.006097561	0.004662005
ARID1A	8289	TP53	7157	0.401430031	0.003658537	0.064102564
ARID1A	8289	VCAN	1462	0.017364658	0.029268293	0.012820513
ASPM	259266	MAPRE1	22919	0.004048583	0.004694836	0.001104972
ASPM	259266	PFDN4	5203	0.082995951	0.008215962	0.001104972
ASPM	259266	SMARCA4	6597	0.0951417	0.009389671	0.00441989
ASPM	259266	SRC	6714	0.174089069	0.03286385	0.015469613
ASPM	259266	SUPT5H	6829	0.006072874	0.005868545	0.007734807
ASPM	259266	TP53	7157	0.486842105	0.003521127	0.007734807

ATM	472	BMP7	655	0.030462185	0.01905972	0.003649635
ATM	472	BMPR2	659	0.026260504	0.002541296	0.001216545
ATM	472	COL1A2	1278	0.035714286	0.07750953	0.01216545
ATM	472	NCOA6	23054	0.116596639	0.001270648	0.00973236
ATM	472	NEURL2	140825	0.033613445	0.001270648	0.001216545
ATM	472	NRAS	4893	0.005252101	0.001270648	0.00729927
ATM	472	PFDN4	5203	0.022058824	0.01905972	0.00243309
ATM	472	RAE1	8480	0.014705882	0.024142313	0.01216545
ATM	472	RBL1	5933	0.046218487	0.001270648	0.00729927
ATM	472	SMARCA4	6597	0.18697479	0.030495553	0.054744526
ATM	472	SRC	6714	0.182773109	0.049555273	0.091240876
ATM	472	SUPT5H	6829	0.077731092	0.011435832	0.023114355
ATM	472	TP53	7157	0.454831933	0.082592122	0.200729927
ATM	472	UBE2V1	7335	0.00105042	0.027954257	0.01703163
ATM	472	VCAN	1462	0.014705882	0.017789072	0.054744526
BMPR2	659	ASPM	259266	0.01187905	0.004968944	0.00844773
BMPR2	659	BMP7	655	0.60475162	0.151552795	0.005279831
BMPR2	659	CASP8	841	0.016198704	0.001242236	0.001055966
BMPR2	659	COL1A2	1278	0.082073434	0.048447205	0.001055966
BMPR2	659	COL5A1	1289	0.001079914	0.013664596	0.006335797
BMPR2	659	FZD3	7976	0.001079914	0.028571429	0.066525871
BMPR2	659	MAPRE1	22919	0.001079914	0.001242236	0.004223865
BMPR2	659	MKI67	4288	0.001079914	0.004968944	0.001055966
BMPR2	659	NEURL2	140825	0.012958963	0.002484472	0.004223865
BMPR2	659	NRAS	4893	0.008639309	0.004968944	0.002111932
BMPR2	659	PFDN4	5203	0.098272138	0.016149068	0.015839493

BMPR2	659	RAE1	8480	0.004319654	0.026086957	0.013727561
BMPR2	659	RBL1	5933	0.062634989	0.004968944	0.033790919
BMPR2	659	SMARCA4	6597	0.075593952	0.021118012	0.033790919
BMPR2	659	SRC	6714	0.193304536	0.053416149	0.110876452
BMPR2	659	SUPT5H	6829	0.018358531	0.013664596	0.010559662
BMPR2	659	TOMM34	10953	0.02699784	0.016149068	0.064413939
BMPR2	659	TP53	7157	0.38336933	0.014906832	0.174234424
BMPR2	659	UBE2V1	7335	0.001079914	0.014906832	0.098204857
BMPR2	659	VCAN	1462	0.008639309	0.011180124	0.009503696
BRAF	673	ASPM	259266	0.010288066	0.006510417	0.003325942
BRAF	673	BMP7	655	0.024691358	0.01953125	0.001108647
BRAF	673	COL1A2	1278	0.052469136	0.0390625	0.007760532
BRAF	673	COL5A1	1289	0.001028807	0.014322917	0.006651885
BRAF	673	NRAS	4893	0.014403292	0.002604167	0.00443459
BRAF	673	PFDN4	5203	0.043209877	0.014322917	0.042128603
BRAF	673	RAE1	8480	0.001028807	0.028645833	0.005543237
BRAF	673	SMARCA4	6597	0.043209877	0.022135417	0.022172949
BRAF	673	SRC	6714	0.232510288	0.0859375	0.129711752
BRAF	673	SUPT5H	6829	0.016460905	0.010416667	0.001108647
BRAF	673	TOMM34	10953	0.013374486	0.00390625	0.012195122
BRAF	673	TP53	7157	0.439300412	0.010416667	0.279379157
BRAF	673	VCAN	1462	0.022633745	0.013020833	0.044345898
CASP8	841	BMP7	655	0.035864979	0.020565553	0.002531646
CASP8	841	BMPR2	659	0.004219409	0.001285347	0.001265823
CASP8	841	COL1A2	1278	0.132911392	0.053984576	0.002531646
CASP8	841	COL5A1	1289	0.003164557	0.082262211	0.007594937

CASP8	841	NEURL2	140825	0.083333333	0.008997429	0.002531646
CASP8	841	NRAS	4893	0.006329114	0.003856041	0.001265823
CASP8	841	PCDH17	27253	0.053797468	0.002570694	0.039240506
CASP8	841	RAE1	8480	0.007383966	0.019280206	0.013924051
CASP8	841	SMARCA4	6597	0.116033755	0.0218509	0.064556962
CASP8	841	SRC	6714	0.165611814	0.033419023	0.041772152
CASP8	841	SUPT5H	6829	0.007383966	0.005141388	0.005063291
CASP8	841	TOMM34	10953	0.037974684	0.007712082	0.036708861
CASP8	841	TP53	7157	0.33649789	0.062982005	0.274683544
CASP8	841	TRRAP	8295	0.003164557	0.002570694	0.008860759
CASP8	841	UBE2V1	7335	0.001054852	0.0218509	0.02278481
CASP8	841	VCAN	1462	0.017932489	0.0218509	0.06835443
CASP8	841	WWOX	51741	0.014767932	0.002570694	0.013924051
CEP250	11190	COL1A2	1278	0.068930041	0.012318029	0.002259887
CEP250	11190	MAPRE1	22919	0.00308642	0.001119821	0.014689266
CEP250	11190	PFDN4	5203	0.076131687	0.012318029	0.002259887
CEP250	11190	RAE1	8480	0.001028807	0.002239642	0.003389831
CEP250	11190	SRC	6714	0.222222222	0.006718925	0.025988701
CEP250	11190	SUPT5H	6829	0.012345679	0.001119821	0.002259887
CEP250	11190	TOMM34	10953	0.00308642	0.007838746	0.04519774
CEP250	11190	TP53	7157	0.403292181	0.001119821	0.006779661
CEP250	11190	VCAN	1462	0.017489712	0.010078387	0.003389831
COL1A2	1278	RAE1	8480	0.002002002	0.021634615	0.001054852
COL1A2	1278	RBL1	5933	0.041041041	0.002403846	0.009493671
COL1A2	1278	SMARCA4	6597	0.023023023	0.013221154	0.004219409
COL1A2	1278	SRC	6714	0.206206206	0.045673077	0.109704641

COL1A2	1278	SUPT5H	6829	0.004004004	0.004807692	0.002109705
COL1A2	1278	TOMM34	10953	0.017017017	0.008413462	0.001054852
COL1A2	1278	TP53	7157	0.389389389	0.002403846	0.183544304
COL1A2	1278	UBE2V1	7335	0.003003003	0.007211538	0.008438819
DOCK2	1794	BMP7	655	0.017060367	0.048780488	0.01308615
DOCK2	1794	BMPR2	659	0.01312336	0.020539153	0.005452563
DOCK2	1794	COL1A2	1278	0.044619423	0.075738126	0.025081788
DOCK2	1794	MAPRE1	22919	0.003937008	0.016688062	0.139585605
DOCK2	1794	NCOA6	23054	0.05511811	0.005134788	0.006543075
DOCK2	1794	NRAS	4893	0.015748031	0.001283697	0.01308615
DOCK2	1794	PCDH17	27253	0.028871391	0.005134788	0.01308615
DOCK2	1794	PFDN4	5203	0.049868766	0.023106547	0.014176663
DOCK2	1794	RAE1	8480	0.009186352	0.028241335	0.117775354
DOCK2	1794	RBL1	5933	0.06824147	0.002567394	0.001090513
DOCK2	1794	SMARCA4	6597	0.094488189	0.053915276	0.017448201
DOCK2	1794	SRC	6714	0.208661417	0.083440308	0.119956379
DOCK2	1794	SUPT5H	6829	0.00656168	0.006418485	0.00436205
DOCK2	1794	TOMM34	10953	0.036745407	0.011553273	0.020719738
DOCK2	1794	TP53	7157	0.290026247	0.030808729	0.173391494
DOCK2	1794	UBE2V1	7335	0.00656168	0.023106547	0.033805889
DOCK2	1794	VCAN	1462	0.032808399	0.050064185	0.050163577
DYNC1H1	1778	ASPM	259266	0.007575758	0.002525253	0.012074643
DYNC1H1	1778	CASP8	841	0.024891775	0.001262626	0.001097695
DYNC1H1	1778	COL1A2	1278	0.079004329	0.045454545	0.008781559
DYNC1H1	1778	COL5A1	1289	0.002164502	0.018939394	0.014270033
DYNC1H1	1778	MAPRE1	22919	0.001082251	0.046717172	0.1701427

DYNC1H1	1778	NEURL2	140825	0.020562771	0.001262626	0.005488474
DYNC1H1	1778	PCDH17	27253	0.04004329	0.001262626	0.013172338
DYNC1H1	1778	PFDN4	5203	0.069264069	0.098484848	0.024149286
DYNC1H1	1778	RAE1	8480	0.002164502	0.032828283	0.075740944
DYNC1H1	1778	SMARCA4	6597	0.031385281	0.017676768	0.004390779
DYNC1H1	1778	SRC	6714	0.273809524	0.039141414	0.10428101
DYNC1H1	1778	SUPT5H	6829	0.011904762	0.006313131	0.00219539
DYNC1H1	1778	TOMM34	10953	0.003246753	0.015151515	0.072447859
DYNC1H1	1778	TP53	7157	0.340909091	0.01010101	0.071350165
DYNC1H1	1778	VCAN	1462	0.016233766	0.025252525	0.027442371
EGFR	1956	ASPM	259266	0.025136612	0.005882353	0.01430143
EGFR	1956	BMP7	655	0.018579235	0.023529412	0.00110011
EGFR	1956	COL1A2	1278	0.133333333	0.076470588	0.04070407
EGFR	1956	COL5A1	1289	0.001092896	0.025882353	0.04730473
EGFR	1956	MAPRE1	22919	0.001092896	0.009411765	0.00110011
EGFR	1956	NRAS	4893	0.002185792	0.004705882	0.00440044
EGFR	1956	PCDH17	27253	0.033879781	0.004705882	0.00440044
EGFR	1956	PFDN4	5203	0.073224044	0.025882353	0.00990099
EGFR	1956	PTPN1	5770	0.101639344	0.005882353	0.02530253
EGFR	1956	RAE1	8480	0.007650273	0.024705882	0.00990099
EGFR	1956	RBL1	5933	0.049180328	0.007058824	0.03740374
EGFR	1956	SMARCA4	6597	0.061202186	0.014117647	0.01870187
EGFR	1956	SRC	6714	0.194535519	0.045882353	0.137513751
EGFR	1956	SUPT5H	6829	0.006557377	0.007058824	0.00330033
EGFR	1956	TOMM34	10953	0.019672131	0.015294118	0.00660066
EGFR	1956	TP53	7157	0.365027322	0.028235294	0.206820682

EGFR	1956	UBE2V1	7335	0.002185792	0.030588235	0.0330033
EGFR	1956	VCAN	1462	0.09726776	0.014117647	0.02970297
EGFR	1956	WWOX	51741	0.057923497	0.005882353	0.00220022
EP300	2033	ASPM	259266	0.007291667	0.002358491	0.004504505
EP300	2033	COL1A2	1278	0.025	0.051886792	0.018018018
EP300	2033	MAPRE1	22919	0.001041667	0.011792453	0.007882883
EP300	2033	NCOA6	23054	0.161458333	0.011792453	0.021396396
EP300	2033	NRAS	4893	0.005208333	0.007075472	0.002252252
EP300	2033	PCDH17	27253	0.03125	0.001179245	0.006756757
EP300	2033	PFDN4	5203	0.00625	0.018867925	0.003378378
EP300	2033	RAE1	8480	0.019791667	0.023584906	0.01463964
EP300	2033	RBL1	5933	0.090625	0.037735849	0.024774775
EP300	2033	SMARCA4	6597	0.105208333	0.008254717	0.064189189
EP300	2033	SRC	6714	0.205208333	0.031839623	0.293918919
EP300	2033	TOMM34	10953	0.014583333	0.010613208	0.003378378
EP300	2033	TP53	7157	0.301041667	0.035377358	0.074324324
EP300	2033	UBE2V1	7335	0.001041667	0.036556604	0.005630631
EP300	2033	VCAN	1462	0.010416667	0.029481132	0.024774775
FBXW7	55294	ASPM	259266	0.008247423	0.003508772	0.015116279
FBXW7	55294	COL1A2	1278	0.060824742	0.07251462	0.015116279
FBXW7	55294	NCOA6	23054	0.04742268	0.001169591	0.010465116
FBXW7	55294	NEURL2	140825	0.025773196	0.001169591	0.002325581
FBXW7	55294	NRAS	4893	0.003092784	0.003508772	0.003488372
FBXW7	55294	PCDH17	27253	0.045360825	0.002339181	0.013953488
FBXW7	55294	PFDN4	5203	0.035051546	0.028070175	0.002325581
FBXW7	55294	RAE1	8480	0.001030928	0.031578947	0.003488372

FBXW7	55294	RBL1	5933	0.150515464	0.005847953	0.06627907
FBXW7	55294	SMARCA4	6597	0.146391753	0.023391813	0.177906977
FBXW7	55294	SRC	6714	0.222680412	0.05380117	0.129069767
FBXW7	55294	SUPT5H	6829	0.032989691	0.021052632	0.022093023
FBXW7	55294	TP53	7157	0.384536082	0.012865497	0.069767442
FBXW7	55294	UBE2V1	7335	0.001030928	0.026900585	0.002325581
FBXW7	55294	VCAN	1462	0.011340206	0.043274854	0.025581395
FLNB	2317	ASPM	259266	0.021327014	0.012850467	0.016326531
FLNB	2317	BMP7	655	0.033175355	0.02453271	0.002040816
FLNB	2317	COL1A2	1278	0.082938389	0.061915888	0.003061224
FLNB	2317	MAPRE1	22919	0.003554502	0.009345794	0.004081633
FLNB	2317	MKI67	4288	0.005924171	0.005841121	0.002040816
FLNB	2317	PFDN4	5203	0.036729858	0.014018692	0.026530612
FLNB	2317	RAE1	8480	0.007109005	0.054906542	0.006122449
FLNB	2317	SMARCA4	6597	0.077014218	0.029205607	0.024489796
FLNB	2317	SRC	6714	0.152843602	0.043224299	0.196938776
FLNB	2317	SUPT5H	6829	0.008293839	0.011682243	0.006122449
FLNB	2317	TOMM34	10953	0.028436019	0.005841121	0.015306122
FLNB	2317	TP53	7157	0.347156398	0.01635514	0.35
FLNB	2317	UBE2V1	7335	0.003554502	0.012850467	0.069387755
FLNB	2317	VCAN	1462	0.084123223	0.030373832	0.030612245
FLNB	2317	WWOX	51741	0.013033175	0.004672897	0.007142857
GRIA1	2890	ASPM	259266	0.00996264	0.012971698	0.020562771
GRIA1	2890	BMP7	655	0.02615193	0.054245283	0.001082251
GRIA1	2890	BMPR2	659	0.00622665	0.005896226	0.001082251
GRIA1	2890	COL1A2	1278	0.059775841	0.070754717	0.021645022

GRIA1	2890	COL5A1	1289	0.00249066	0.029481132	0.035714286
GRIA1	2890	FZD3	7976	0.00249066	0.058962264	0.090909091
GRIA1	2890	MAPRE1	22919	0.00249066	0.010613208	0.004329004
GRIA1	2890	NCOA6	23054	0.02864259	0.004716981	0.03030303
GRIA1	2890	NEURL2	140825	0.066002491	0.003537736	0.001082251
GRIA1	2890	NRAS	4893	0.00622665	0.005896226	0.001082251
GRIA1	2890	PCDH17	27253	0.03486924	0.002358491	0.011904762
GRIA1	2890	PFDN4	5203	0.01743462	0.018867925	0.037878788
GRIA1	2890	RAE1	8480	0.00747198	0.030660377	0.008658009
GRIA1	2890	RBL1	5933	0.068493151	0.027122642	0.029220779
GRIA1	2890	SMARCA4	6597	0.113325031	0.051886792	0.055194805
GRIA1	2890	SRC	6714	0.175591532	0.029481132	0.178571429
GRIA1	2890	SUPT5H	6829	0.00996264	0.016509434	0.00974026
GRIA1	2890	TOMM34	10953	0.03362391	0.007075472	0.027056277
GRIA1	2890	TP53	7157	0.320049813	0.025943396	0.196969697
GRIA1	2890	TRRAP	8295	0.00622665	0.001179245	0.003246753
GRIA1	2890	UBE2V1	7335	0.00996264	0.04009434	0.045454545
GRIA1	2890	VCAN	1462	0.01494396	0.034198113	0.07034632
HCLS1	3059	ASPM	259266	0.011320755	0.005284016	0.004255319
HCLS1	3059	BMP7	655	0.013836478	0.027741083	0.00212766
HCLS1	3059	COL1A2	1278	0.040251572	0.066050198	0.013829787
HCLS1	3059	MAPRE1	22919	0.001257862	0.025099075	0.024468085
HCLS1	3059	NCOA6	23054	0.07672956	0.001321004	0.003191489
HCLS1	3059	NEURL2	140825	0.050314465	0.001321004	0.004255319
HCLS1	3059	NRAS	4893	0.010062893	0.001321004	0.011702128
HCLS1	3059	PCDH17	27253	0.023899371	0.002642008	0.008510638

HCLS1	3059	PFDN4	5203	0.044025157	0.023778071	0.011702128
HCLS1	3059	RAE1	8480	0.010062893	0.036988111	0.014893617
HCLS1	3059	RHPN2	85415	0.008805031	0.001321004	0.00106383
HCLS1	3059	SMARCA4	6597	0.094339623	0.05680317	0.023404255
HCLS1	3059	SRC	6714	0.300628931	0.077939234	0.092553191
HCLS1	3059	SUPT5H	6829	0.006289308	0.009247028	0.004255319
HCLS1	3059	TOMM34	10953	0.046540881	0.01321004	0.04787234
HCLS1	3059	TP53	7157	0.304402516	0.019815059	0.165957447
HCLS1	3059	UBE2V1	7335	0.001257862	0.023778071	0.023404255
HCLS1	3059	VCAN	1462	0.012578616	0.043593131	0.024468085
HNRNPUL1	11100	COL1A2	1278	0.051671733	0.041457286	0.004132231
HNRNPUL1	11100	COL5A1	1289	0.004052685	0.01758794	0.002066116
HNRNPUL1	11100	FZD3	7976	0.001013171	0.016331658	0.015495868
HNRNPUL1	11100	PCDH17	27253	0.060790274	0.002512563	0.005165289
HNRNPUL1	11100	PFDN4	5203	0.00810537	0.011306533	0.001033058
HNRNPUL1	11100	RAE1	8480	0.05775076	0.022613065	0.128099174
HNRNPUL1	11100	SMARCA4	6597	0.069908815	0.007537688	0.009297521
HNRNPUL1	11100	SRC	6714	0.192502533	0.023869347	0.026859504
HNRNPUL1	11100	SUPT5H	6829	0.017223911	0.001256281	0.003099174
HNRNPUL1	11100	TP53	7157	0.574468085	0.00879397	0.073347107
HNRNPUL1	11100	UBE2V1	7335	0.002026342	0.01758794	0.002066116
HNRNPUL1	11100	VCAN	1462	0.018237082	0.022613065	0.027892562
ID1	3397	ASPM	259266	0.007268951	0.01396648	0.002932551
ID1	3397	COL1A2	1278	0.034267913	0.05027933	0.014662757
ID1	3397	NRAS	4893	0.007268951	0.002793296	0.002932551
ID1	3397	RAE1	8480	0.007268951	0.01396648	0.017595308

ID1	3397	RBL1	5933	0.040498442	0.041899441	0.020527859
ID1	3397	SMARCA4	6597	0.092419522	0.016759777	0.008797654
ID1	3397	SRC	6714	0.188992731	0.069832402	0.117302053
ID1	3397	SUPT5H	6829	0.00623053	0.008379888	0.005865103
ID1	3397	TOMM34	10953	0.041536864	0.008379888	0.008797654
ID1	3397	TP53	7157	0.394600208	0.019553073	0.114369501
ID1	3397	VCAN	1462	0.018691589	0.019553073	0.04398827
IGF2R	3482	ASPM	259266	0.010341262	0.003754693	0.003194888
IGF2R	3482	BMP7	655	0.032057911	0.026282854	0.002129925
IGF2R	3482	COL1A2	1278	0.066184074	0.023779725	0.006389776
IGF2R	3482	COL5A1	1289	0.001034126	0.003754693	0.027689031
IGF2R	3482	MAPRE1	22919	0.010341262	0.003754693	0.002129925
IGF2R	3482	NCOA6	23054	0.077559462	0.001251564	0.001064963
IGF2R	3482	PFDN4	5203	0.007238883	0.018773467	0.010649627
IGF2R	3482	RAE1	8480	0.001034126	0.01126408	0.002129925
IGF2R	3482	RBL1	5933	0.189245088	0.010012516	0.006389776
IGF2R	3482	SMARCA4	6597	0.097207859	0.020025031	0.02342918
IGF2R	3482	SRC	6714	0.160289555	0.026282854	0.057507987
IGF2R	3482	SUPT5H	6829	0.012409514	0.005006258	0.006389776
IGF2R	3482	TOMM34	10953	0.021716649	0.005006258	0.018104366
IGF2R	3482	TP53	7157	0.351602896	0.023779725	0.375931842
IGF2R	3482	UBE2V1	7335	0.003102378	0.023779725	0.047923323
IGF2R	3482	VCAN	1462	0.021716649	0.020025031	0.018104366
IQGAP2	10788	ASPM	259266	0.011857708	0.016369048	0.003118503
IQGAP2	10788	BMP7	655	0.040843215	0.026785714	0.009355509
IQGAP2	10788	BMPR2	659	0.005270092	0.004464286	0.001039501

IQGAP2	10788	COL1A2	1278	0.048748353	0.095238095	0.041580042
IQGAP2	10788	MKI67	4288	0.001317523	0.00297619	0.004158004
IQGAP2	10788	NRAS	4893	0.019762846	0.007440476	0.005197505
IQGAP2	10788	PCDH17	27253	0.027667984	0.00297619	0.008316008
IQGAP2	10788	PFDN4	5203	0.075098814	0.044642857	0.011434511
IQGAP2	10788	RAE1	8480	0.011857708	0.050595238	0.09043659
IQGAP2	10788	RBL1	5933	0.046113307	0.005952381	0.017671518
IQGAP2	10788	SMARCA4	6597	0.093544137	0.044642857	0.018711019
IQGAP2	10788	SRC	6714	0.202898551	0.044642857	0.230769231
IQGAP2	10788	SUPT5H	6829	0.036890646	0.032738095	0.012474012
IQGAP2	10788	TOMM34	10953	0.040843215	0.014880952	0.016632017
IQGAP2	10788	TP53	7157	0.324110672	0.029761905	0.171517672
IQGAP2	10788	UBE2V1	7335	0.003952569	0.025297619	0.077962578
IQGAP2	10788	VCAN	1462	0.019762846	0.055059524	0.034303534
ITPR3	3710	ANK1	286	0.966084275	0.017220172	0.004201681
ITPR3	3710	ASPM	259266	0.003083248	0.001230012	0.00105042
ITPR3	3710	COL1A2	1278	0.322713258	0.052890529	0.006302521
ITPR3	3710	COL5A1	1289	0.003083248	0.018450185	0.017857143
ITPR3	3710	MAPRE1	22919	0.007194245	0.028290283	0.00105042
ITPR3	3710	PCDH17	27253	0.038026721	0.001230012	0.012605042
ITPR3	3710	RAE1	8480	0.006166495	0.019680197	0.008403361
ITPR3	3710	RBL1	5933	0.04008222	0.009840098	0.003151261
ITPR3	3710	SMARCA4	6597	0.036998972	0.008610086	0.015756303
ITPR3	3710	SRC	6714	0.142857143	0.041820418	0.101890756
ITPR3	3710	SUPT5H	6829	0.004110997	0.006150062	0.009453782
ITPR3	3710	TOMM34	10953	0.019527235	0.011070111	0.036764706

ITPR3	3710	TP53	7157	0.344295992	0.006150062	0.069327731
ITPR3	3710	VCAN	1462	0.284686536	0.011070111	0.008403361
KRAS	3845	ASPM	259266	0.011351909	0.010895884	0.01194354
KRAS	3845	COL1A2	1278	0.097007224	0.069007264	0.01194354
KRAS	3845	COL5A1	1289	0.005159959	0.032687651	0.021715527
KRAS	3845	MAPRE1	22919	0.003095975	0.007263923	0.007600434
KRAS	3845	NRAS	4893	0.080495356	0.002421308	0.010857763
KRAS	3845	PCDH17	27253	0.026831785	0.003631961	0.009771987
KRAS	3845	PFDN4	5203	0.019607843	0.026634383	0.013029316
KRAS	3845	RAE1	8480	0.012383901	0.027845036	0.014115092
KRAS	3845	RBL1	5933	0.07120743	0.010895884	0.022801303
KRAS	3845	SMARCA4	6597	0.057791538	0.054479419	0.028230185
KRAS	3845	SRC	6714	0.235294118	0.055690073	0.208469055
KRAS	3845	SUPT5H	6829	0.011351909	0.01937046	0.003257329
KRAS	3845	TOMM34	10953	0.035087719	0.016949153	0.026058632
KRAS	3845	TP53	7157	0.308565531	0.012106538	0.250814332
KRAS	3845	VCAN	1462	0.044375645	0.039951574	0.034744843
KRAS	3845	WWOX	51741	0.030959752	0.003631961	0.005428882
LAMA1	284217	ASPM	259266	0.008333333	0.012805588	0.045647558
LAMA1	284217	BMP7	655	0.016666667	0.037252619	0.001061571
LAMA1	284217	CASP8	841	0.077380952	0.001164144	0.001061571
LAMA1	284217	COL1A2	1278	0.304761905	0.088474971	0.008492569
LAMA1	284217	COL5A1	1289	0.010714286	0.030267753	0.010615711
LAMA1	284217	MAPRE1	22919	0.002380952	0.010477299	0.006369427
LAMA1	284217	MKI67	4288	0.001190476	0.009313155	0.001061571
LAMA1	284217	NCOA6	23054	0.041666667	0.001164144	0.004246285

LAMA1	284217	NRAS	4893	0.011904762	0.005820722	0.005307856
LAMA1	284217	PCDH17	27253	0.008333333	0.003492433	0.00955414
LAMA1	284217	PFDN4	5203	0.010714286	0.046565774	0.167728238
LAMA1	284217	RAE1	8480	0.00952381	0.022118743	0.005307856
LAMA1	284217	RBL1	5933	0.079761905	0.004656577	0.020169851
LAMA1	284217	SMARCA4	6597	0.113095238	0.036088475	0.047770701
LAMA1	284217	SRC	6714	0.13452381	0.016298021	0.02866242
LAMA1	284217	SUPT5H	6829	0.013095238	0.011641444	0.002123142
LAMA1	284217	TOMM34	10953	0.027380952	0.00814901	0.013800425
LAMA1	284217	TP53	7157	0.338095238	0.055878929	0.369426752
LAMA1	284217	TRRAP	8295	0.020238095	0.006984866	0.007430998
LAMA1	284217	UBE2V1	7335	0.003571429	0.031431898	0.045647558
LAMA1	284217	VCAN	1462	0.051190476	0.036088475	0.105095541
LAMA1	284217	WWOX	51741	0.032142857	0.001164144	0.01910828
LIFR	3977	ASPM	259266	0.00968523	0.004076087	0.013172338
LIFR	3977	BMP7	655	0.010895884	0.07201087	0.008781559
LIFR	3977	BMPR2	659	0.003631961	0.004076087	0.001097695
LIFR	3977	CASP8	841	0.076271186	0.002717391	0.007683864
LIFR	3977	COL1A2	1278	0.050847458	0.080163043	0.053787047
LIFR	3977	COL5A1	1289	0.001210654	0.046195652	0.076838639
LIFR	3977	FZD3	7976	0.002421308	0.141304348	0.124039517
LIFR	3977	IGF2R	3482	0.003631961	0.001358696	0.001097695
LIFR	3977	ITPR3	3710	0.001210654	0.005434783	0.001097695
LIFR	3977	MAPRE1	22919	0.004842615	0.008152174	0.004390779
LIFR	3977	NCOA6	23054	0.094430993	0.001358696	0.00219539
LIFR	3977	NRAS	4893	0.014527845	0.004076087	0.006586169

LIFR	3977	PCDH17	27253	0.014527845	0.001358696	0.015367728
LIFR	3977	PFDN4	5203	0.033898305	0.03125	0.004390779
LIFR	3977	RAE1	8480	0.007263923	0.024456522	0.03183315
LIFR	3977	RBL1	5933	0.082324455	0.005434783	0.004390779
LIFR	3977	SMARCA4	6597	0.118644068	0.035326087	0.03402854
LIFR	3977	SRC	6714	0.295399516	0.048913043	0.296377607
LIFR	3977	SUPT5H	6829	0.025423729	0.008152174	0.005488474
LIFR	3977	TOMM34	10953	0.031476998	0.008152174	0.07025247
LIFR	3977	TP53	7157	0.308716707	0.042119565	0.225027442
LIFR	3977	VCAN	1462	0.012106538	0.057065217	0.043907794
LRP1B	53353	ASPM	259266	0.005144033	0.002506266	0.018691589
LRP1B	53353	PFDN4	5203	0.013374486	0.040100251	0.009345794
LRP1B	53353	RBL1	5933	0.051440329	0.005012531	0.037383178
LRP1B	53353	SMARCA4	6597	0.053497942	0.020050125	0.08411215
LRP1B	53353	SRC	6714	0.158436214	0.065162907	0.08411215
LRP1B	53353	SUPT5H	6829	0.010288066	0.010025063	0.028037383
LRP1B	53353	TP53	7157	0.364197531	0.020050125	0.168224299
LRP1B	53353	VCAN	1462	0.019547325	0.022556391	0.009345794
LRP2	4036	NCOA6	23054	0.033299697	0.001111111	0.001023541
LRP2	4036	RBL1	5933	0.073662967	0.002222222	0.004094166
LRP2	4036	SMARCA4	6597	0.091826438	0.007777778	0.004094166
LRP2	4036	SRC	6714	0.165489405	0.016666667	0.006141249
LRP2	4036	TP53	7157	0.396569122	0.021111111	0.020470829
MAP3K4	4216	ASPM	259266	0.007246377	0.003875969	0.001046025
MAP3K4	4216	BMP7	655	0.022774327	0.016795866	0.001046025
MAP3K4	4216	COL1A2	1278	0.054865424	0.047803618	0.008368201

MAP3K4	4216	MAPRE1	22919	0.010351967	0.002583979	0.073221757
MAP3K4	4216	NEURL2	140825	0.039337474	0.00129199	0.00209205
MAP3K4	4216	NRAS	4893	0.044513458	0.003875969	0.001046025
MAP3K4	4216	PFDN4	5203	0.051759834	0.029715762	0.006276151
MAP3K4	4216	RAE1	8480	0.007246377	0.020671835	0.020920502
MAP3K4	4216	SMARCA4	6597	0.062111801	0.015503876	0.009414226
MAP3K4	4216	SRC	6714	0.238095238	0.07751938	0.072175732
MAP3K4	4216	SUPT5H	6829	0.005175983	0.005167959	0.001046025
MAP3K4	4216	TOMM34	10953	0.02484472	0.009043928	0.00209205
MAP3K4	4216	TP53	7157	0.414078675	0.006459948	0.04707113
MAP3K4	4216	UBE2V1	7335	0.001035197	0.015503876	0.024058577
MAP3K4	4216	VCAN	1462	0.019668737	0.015503876	0.010460251
MAPRE1	22919	ASPM	259266	0.012244898	0.005841121	0.004287245
MAPRE1	22919	COL1A2	1278	0.064285714	0.036214953	0.001071811
MAPRE1	22919	RAE1	8480	0.010204082	0.005841121	0.235798499
MAPRE1	22919	SMARCA4	6597	0.06122449	0.074766355	0.004287245
MAPRE1	22919	SRC	6714	0.203061224	0.029205607	0.01607717
MAPRE1	22919	SUPT5H	6829	0.009183673	0.01635514	0.003215434
MAPRE1	22919	TP53	7157	0.495918367	0.010514019	0.019292605
MAPRE1	22919	VCAN	1462	0.023469388	0.010514019	0.003215434
MKI67	4288	COL1A2	1278	0.056508577	0.036553525	0.007314525
MKI67	4288	COL5A1	1289	0.002018163	0.018276762	0.010449321
MKI67	4288	SMARCA4	6597	0.106962664	0.005221932	0.012539185
MKI67	4288	SRC	6714	0.13925328	0.03002611	0.183908046
MKI67	4288	SUPT5H	6829	0.01715439	0.006527415	0.003134796
MKI67	4288	TP53	7157	0.336024218	0.001305483	0.004179728

MKI67	4288	VCAN	1462	0.018163471	0.005221932	0.004179728
NCOA6	23054	ASPM	259266	0.008088979	0.001164144	0.002364066
NCOA6	23054	COL1A2	1278	0.037411527	0.022118743	0.005910165
NCOA6	23054	COL5A1	1289	0.002022245	0.013969732	0.003546099
NCOA6	23054	NRAS	4893	0.004044489	0.001164144	0.001182033
NCOA6	23054	RBL1	5933	0.023255814	0.002328289	0.011820331
NCOA6	23054	SMARCA4	6597	0.089989889	0.020954598	0.073286052
NCOA6	23054	SRC	6714	0.193124368	0.015133877	0.041371158
NCOA6	23054	SUPT5H	6829	0.062689585	0.00814901	0.011820331
NCOA6	23054	TP53	7157	0.46107179	0.025611176	0.115839243
NCOA6	23054	VCAN	1462	0.026289181	0.009313155	0.022458629
NFATC2	4773	ASPM	259266	0.01975052	0.005959476	0.006703911
NFATC2	4773	BMP7	655	0.020790021	0.017878427	0.005586592
NFATC2	4773	COL1A2	1278	0.038461538	0.075089392	0.005586592
NFATC2	4773	COL5A1	1289	0.001039501	0.020262217	0.013407821
NFATC2	4773	PCDH17	27253	0.041580042	0.005959476	0.010055866
NFATC2	4773	PFDN4	5203	0.021829522	0.025029797	0.005586592
NFATC2	4773	RAE1	8480	0.007276507	0.019070322	0.027932961
NFATC2	4773	RBL1	5933	0.029106029	0.007151371	0.010055866
NFATC2	4773	SMARCA4	6597	0.092515593	0.030989273	0.14972067
NFATC2	4773	SRC	6714	0.206860707	0.034564958	0.141899441
NFATC2	4773	SUPT5H	6829	0.01975052	0.00476758	0.003351955
NFATC2	4773	TP53	7157	0.381496881	0.023837902	0.316201117
NFATC2	4773	UBE2V1	7335	0.001039501	0.017878427	0.011173184
NFATC2	4773	VCAN	1462	0.014553015	0.033373063	0.082681564
NRAS	4893	ASPM	259266	0.015321757	0.001193317	0.00422833

NRAS	4893	COL1A2	1278	0.061287028	0.033412888	0.00845666
NRAS	4893	COL5A1	1289	0.00102145	0.015513126	0.012684989
NRAS	4893	MAPRE1	22919	0.005107252	0.007159905	0.011627907
NRAS	4893	PCDH17	27253	0.039836568	0.001193317	0.001057082
NRAS	4893	PFDN4	5203	0.046986721	0.010739857	0.002114165
NRAS	4893	RAE1	8480	0.010214505	0.016706444	0.014799154
NRAS	4893	RBL1	5933	0.041879469	0.007159905	0.003171247
NRAS	4893	SMARCA4	6597	0.04392237	0.066825776	0.019027484
NRAS	4893	SRC	6714	0.19918284	0.03221957	0.115221987
NRAS	4893	SUPT5H	6829	0.010214505	0.026252983	0.006342495
NRAS	4893	TOMM34	10953	0.039836568	0.008353222	0.00845666
NRAS	4893	TP53	7157	0.378958121	0.017899761	0.21987315
NRAS	4893	VCAN	1462	0.017364658	0.022673031	0.013742072
NRAS	4893	WWOX	51741	0.009193054	0.002386635	0.001057082
PFDN4	5203	RAE1	8480	0.012182741	0.004371585	0.039354188
PFDN4	5203	SRC	6714	0.190862944	0.006557377	0.002018163
PFDN4	5203	TOMM34	10953	0.01319797	0.007650273	0.001009082
PFDN4	5203	TP53	7157	0.491370558	0.007650273	0.003027245
PI4KA	5297	ASPM	259266	0.01626898	0.002386635	0.016611296
PI4KA	5297	BMP7	655	0.034707158	0.010739857	0.00110742
PI4KA	5297	COL1A2	1278	0.035791757	0.054892601	0.021040975
PI4KA	5297	COL5A1	1289	0.002169197	0.022673031	0.03875969
PI4KA	5297	MAPRE1	22919	0.004338395	0.013126492	0.014396456
PI4KA	5297	NRAS	4893	0.004338395	0.002386635	0.008859358
PI4KA	5297	PFDN4	5203	0.035791757	0.04176611	0.005537099
PI4KA	5297	RAE1	8480	0.015184382	0.027446301	0.018826135

PI4KA	5297	RBL1	5933	0.045553145	0.00477327	0.012181617
PI4KA	5297	SMARCA4	6597	0.075921909	0.131264916	0.015503876
PI4KA	5297	SRC	6714	0.176789588	0.046539379	0.132890365
PI4KA	5297	SUPT5H	6829	0.006507592	0.010739857	0.006644518
PI4KA	5297	TOMM34	10953	0.011930586	0.014319809	0.016611296
PI4KA	5297	TP53	7157	0.355748373	0.017899761	0.210409745
PI4KA	5297	UBE2V1	7335	0.013015184	0.025059666	0.026578073
PI4KA	5297	VCAN	1462	0.015184382	0.03699284	0.043189369
PI4KA	5297	WWOX	51741	0.034707158	0.002386635	0.00110742
PLAT	5327	ASPM	259266	0.008510638	0.009888752	0.013001083
PLAT	5327	BMP7	655	0.011702128	0.037082818	0.004333694
PLAT	5327	COL1A2	1278	0.2	0.121137206	0.014084507
PLAT	5327	COL5A1	1289	0.006382979	0.038318912	0.017334778
PLAT	5327	FZD3	7976	0.00106383	0.037082818	0.058504875
PLAT	5327	IGF2R	3482	0.043617021	0.002472188	0.02708559
PLAT	5327	NCOA6	23054	0.04893617	0.002472188	0.026002167
PLAT	5327	PCDH17	27253	0.036170213	0.002472188	0.006500542
PLAT	5327	PFDN4	5203	0.011702128	0.019777503	0.004333694
PLAT	5327	RAE1	8480	0.004255319	0.033374536	0.017334778
PLAT	5327	RBL1	5933	0.104255319	0.004944376	0.03900325
PLAT	5327	SMARCA4	6597	0.135106383	0.04697157	0.082340195
PLAT	5327	SRC	6714	0.257446809	0.061804697	0.169014085
PLAT	5327	SUPT5H	6829	0.017021277	0.004944376	0.019501625
PLAT	5327	TOMM34	10953	0.024468085	0.009888752	0.009750813
PLAT	5327	TP53	7157	0.342553191	0.028430161	0.336944745
PLAT	5327	TRRAP	8295	0.006382979	0.003708282	0.003250271

PLAT	5327	UBE2V1	7335	0.007446809	0.024721879	0.154929577
PLAT	5327	VCAN	1462	0.018085106	0.009888752	0.015167931
PLAT	5327	WWOX	51741	0.035106383	0.001236094	0.002166847
PTPN1	5770	ASPM	259266	0.011077543	0.002531646	0.005720824
PTPN1	5770	COL1A2	1278	0.06143001	0.046835443	0.006864989
PTPN1	5770	SMARCA4	6597	0.062437059	0.007594937	0.011441648
PTPN1	5770	SRC	6714	0.249748238	0.063291139	0.070938215
PTPN1	5770	SUPT5H	6829	0.007049345	0.003797468	0.00228833
PTPN1	5770	TOMM34	10953	0.053373615	0.005063291	0.012585812
PTPN1	5770	TP53	7157	0.402819738	0.012658228	0.315789474
PTPN1	5770	UBE2V1	7335	0.001007049	0.008860759	0.001144165
PTPN1	5770	VCAN	1462	0.029204431	0.006329114	0.038901602
RBL1	5933	COL1A2	1278	0.054271357	0.013318535	0.001146789
RBL1	5933	COL5A1	1289	0.001005025	0.002219756	0.003440367
RBL1	5933	SMARCA4	6597	0.115577889	0.008879023	0.047018349
RBL1	5933	SRC	6714	0.248241206	0.008879023	0.028669725
RBL1	5933	SUPT5H	6829	0.012060302	0.001109878	0.017201835
RBL1	5933	TP53	7157	0.468341709	0.004439512	0.029816514
RBL1	5933	VCAN	1462	0.038190955	0.012208657	0.010321101
SMAD4	4089	ASPM	259266	0.006335797	0.004956629	0.012716763
SMAD4	4089	BMP7	655	0.043294615	0.142503098	0.001156069
SMAD4	4089	CASP8	841	0.042238648	0.001239157	0.003468208
SMAD4	4089	COL1A2	1278	0.042238648	0.066914498	0.006936416
SMAD4	4089	COL5A1	1289	0.002111932	0.02850062	0.009248555
SMAD4	4089	FZD3	7976	0.001055966	0.038413879	0.159537572
SMAD4	4089	MAPRE1	22919	0.001055966	0.012391574	0.001156069

SMAD4	4089	NEURL2	140825	0.033790919	0.002478315	0.001156069
SMAD4	4089	NRAS	4893	0.080253432	0.003717472	0.002312139
SMAD4	4089	PFDN4	5203	0.016895459	0.024783147	0.015028902
SMAD4	4089	RAE1	8480	0.009503696	0.022304833	0.005780347
SMAD4	4089	RBL1	5933	0.218585005	0.060718711	0.034682081
SMAD4	4089	SMARCA4	6597	0.120380148	0.037174721	0.269364162
SMAD4	4089	SRC	6714	0.166842661	0.060718711	0.107514451
SMAD4	4089	SUPT5H	6829	0.009503696	0.019826518	0.004624277
SMAD4	4089	TOMM34	10953	0.024287223	0.009913259	0.002312139
SMAD4	4089	TP53	7157	0.337909187	0.068153656	0.097109827
SMAD4	4089	UBE2V1	7335	0.001055966	0.045848823	0.012716763
SMAD4	4089	VCAN	1462	0.012671595	0.033457249	0.021965318
SMARCA4	6597	COL5A1	1289	0.003006012	0.007667032	0.001086957
SMARCA4	6597	PFDN4	5203	0.011022044	0.004381161	0.075
SMARCA4	6597	RAE1	8480	0.002004008	0.010952903	0.007608696
SMARCA4	6597	SRC	6714	0.265531062	0.007667032	0.067391304
SMARCA4	6597	SUPT5H	6829	0.01002004	0.003285871	0.002173913
SMARCA4	6597	TP53	7157	0.471943888	0.003285871	0.002173913
SMARCA4	6597	VCAN	1462	0.034068136	0.007667032	0.002173913
SRC	6714	ASPM	259266	0.011178862	0.011961722	0.002234637
SRC	6714	COL1A2	1278	0.036585366	0.15430622	0.026815642
SRC	6714	MAPRE1	22919	0.008130081	0.005980861	0.018994413
SRC	6714	PFDN4	5203	0.011178862	0.007177033	0.001117318
SRC	6714	RBL1	5933	0.05995935	0.001196172	0.004469274
SRC	6714	SMARCA4	6597	0.06402439	0.005980861	0.011173184
SRC	6714	TOMM34	10953	0.071138211	0.008373206	0.015642458

SRC	6714	TP53	7157	0.347560976	0.027511962	0.084916201
SRC	6714	VCAN	1462	0.014227642	0.005980861	0.017877095
SUPT5H	6829	COL1A2	1278	0.060913706	0.030162413	0.002155172
SUPT5H	6829	COL5A1	1289	0.001015228	0.012761021	0.001077586
SUPT5H	6829	MAPRE1	22919	0.005076142	0.002320186	0.005387931
SUPT5H	6829	MKI67	4288	0.001015228	0.001160093	0.002155172
SUPT5H	6829	NRAS	4893	0.007106599	0.001160093	0.001077586
SUPT5H	6829	PFDN4	5203	0.014213198	0.015081206	0.011853448
SUPT5H	6829	SMARCA4	6597	0.075126904	0.032482599	0.005387931
SUPT5H	6829	SRC	6714	0.256852792	0.017401392	0.045258621
SUPT5H	6829	TP53	7157	0.379695431	0.005800464	0.020474138
SUPT5H	6829	VCAN	1462	0.01928934	0.017401392	0.009698276
TP53	7157	BMP7	655	0.008032129	0.006841505	0.001144165
TP53	7157	COL1A2	1278	0.054216867	0.019384265	0.001144165
TP53	7157	MAPRE1	22919	0.001004016	0.005701254	0.006864989
TP53	7157	PFDN4	5203	0.011044177	0.007981756	0.00228833
TP53	7157	RAE1	8480	0.004016064	0.01368301	0.006864989
TP53	7157	RBL1	5933	0.027108434	0.034207526	0.005720824
TP53	7157	SMARCA4	6597	0.16064257	0.010262258	0.058352403
TP53	7157	SRC	6714	0.215863454	0.014823261	0.068649886
TP53	7157	SUPT5H	6829	0.025100402	0.007981756	0.010297483
TP53	7157	VCAN	1462	0.019076305	0.009122007	0.258581236
TRRAP	8295	ASPM	259266	0.011144883	0.002424242	0.002229654
TRRAP	8295	COL1A2	1278	0.061803445	0.019393939	0.006688963
TRRAP	8295	COL5A1	1289	0.003039514	0.004848485	0.003344482
TRRAP	8295	FZD3	7976	0.002026342	0.006060606	0.05574136

TRRAP	8295	NRAS	4893	0.001013171	0.002424242	0.001114827
TRRAP	8295	PCDH17	27253	0.028368794	0.001212121	0.001114827
TRRAP	8295	RAE1	8480	0.002026342	0.013333333	0.002229654
TRRAP	8295	RBL1	5933	0.089159068	0.024242424	0.005574136
TRRAP	8295	SMARCA4	6597	0.147922999	0.006060606	0.016722408
TRRAP	8295	SRC	6714	0.20668693	0.018181818	0.057971014
TRRAP	8295	SUPT5H	6829	0.021276596	0.008484848	0.004459309
TRRAP	8295	TP53	7157	0.436676798	0.001212121	0.03567447
TRRAP	8295	UBE2V1	7335	0.002026342	0.00969697	0.003344482
TRRAP	8295	VCAN	1462	0.034447822	0.021818182	0.005574136
VAV1	7409	COL1A2	1278	0.035476718	0.079837618	0.01870187
VAV1	7409	MAPRE1	22919	0.002217295	0.01894452	0.03630363
VAV1	7409	MKI67	4288	0.001108647	0.00405954	0.00110011
VAV1	7409	NRAS	4893	0.017738359	0.01082544	0.02970297
VAV1	7409	PCDH17	27253	0.021064302	0.00270636	0.00660066
VAV1	7409	PFDN4	5203	0.139689579	0.029769959	0.00220022
VAV1	7409	RAE1	8480	0.003325942	0.029769959	0.02970297
VAV1	7409	RBL1	5933	0.034368071	0.00135318	0.00990099
VAV1	7409	SMARCA4	6597	0.095343681	0.025710419	0.01210121
VAV1	7409	SRC	6714	0.238359202	0.070365359	0.121012101
VAV1	7409	SUPT5H	6829	0.034368071	0.01488498	0.00110011
VAV1	7409	TOMM34	10953	0.032150776	0.01217862	0.00990099
VAV1	7409	TP53	7157	0.349223947	0.01894452	0.126512651
VAV1	7409	TRRAP	8295	0.005543237	0.00270636	0.00110011
VAV1	7409	UBE2V1	7335	0.00443459	0.02165088	0.01650165
VAV1	7409	VCAN	1462	0.018847007	0.035182679	0.01540154

VCAN	1462	ASPM	259266	0.005994006	0.001145475	0.005154639
VCAN	1462	RBL1	5933	0.034965035	0.002290951	0.010309278
VCAN	1462	SMARCA4	6597	0.022977023	0.009163803	0.005154639
VCAN	1462	SRC	6714	0.162837163	0.009163803	0.046391753
VCAN	1462	TP53	7157	0.352647353	0.006872852	0.021649485
VCAN	1462	UBE2V1	7335	0.001998002	0.020618557	0.006185567
WWOX	51741	ASPM	259266	0.016096579	0.003759398	0.004489338
WWOX	51741	BMP7	655	0.017102616	0.013784461	0.004489338
WWOX	51741	CASP8	841	0.010060362	0.001253133	0.003367003
WWOX	51741	COL1A2	1278	0.058350101	0.060150376	0.002244669
WWOX	51741	NCOA6	23054	0.025150905	0.001253133	0.002244669
WWOX	51741	RAE1	8480	0.003018109	0.01754386	0.004489338
WWOX	51741	RBL1	5933	0.030181087	0.002506266	0.002244669
WWOX	51741	SMARCA4	6597	0.130784708	0.109022556	0.105499439
WWOX	51741	SRC	6714	0.286720322	0.045112782	0.106621773
WWOX	51741	SUPT5H	6829	0.010060362	0.007518797	0.008978676
WWOX	51741	TOMM34	10953	0.028169014	0.006265664	0.001122334
WWOX	51741	TP53	7157	0.742454728	0.05764411	1
WWOX	51741	VCAN	1462	0.014084507	0.00877193	0.210998878

Table S7 Fitness relationships common in FNs for SKCM with corresponding frequency

Gene 1	Entrez ID	Gene 2	Entrez ID	Frequency_HPRD	Frequency_Humannet	Frequency_PPIwu
ACTN2	88	FLT1	2321	0.005827506	0.018691589	0.040358744
ACTN2	88	GRIN2A	2903	0.002331002	0.028037383	0.071748879
ACTN2	88	HDAC9	9734	0.001165501	0.088785047	0.01793722
ACTN2	88	MDC1	9656	0.002331002	0.03271028	0.004484305

ACTN2	88	TTN	7273	0.006993007	0.056074766	0.062780269
AHNAK	79026	ANK2	287	0.00122399	0.004291845	0.001557632
AHNAK	79026	BRAF	673	0.107711138	0.0472103	0.066978193
AHNAK	79026	CDKN2A	1029	0.283965728	0.17167382	0.024922118
AHNAK	79026	FLT1	2321	0.00122399	0.004291845	0.00623053
AHNAK	79026	GRM8	2918	0.00122399	0.008583691	0.009345794
AHNAK	79026	HDAC9	9734	0.00244798	0.077253219	0.034267913
AHNAK	79026	MDC1	9656	0.024479804	0.051502146	0.001557632
AHNAK	79026	NF1	4763	0.00122399	0.042918455	0.00623053
AHNAK	79026	NRAS	4893	0.00122399	0.008583691	0.010903427
AHNAK	79026	PTEN	5728	0.017135863	0.034334764	0.020249221
AHNAK	79026	ZFHX3	463	0.00122399	0.017167382	0.001557632
ANK1	286	ACTN2	88	0.014893617	0.028368794	0.014285714
ANK1	286	ANK2	287	0.006382979	0.056737589	0.142857143
ANK1	286	FLT1	2321	0.010638298	0.007092199	0.028571429
ANK1	286	GRIN2A	2903	0.010638298	0.063829787	0.157142857
ANK1	286	KDR	3791	0.00212766	0.014184397	0.014285714
ANK1	286	MDC1	9656	0.00212766	0.014184397	0.042857143
ANK1	286	NF1	4763	0.004255319	0.028368794	0.057142857
ANK1	286	PPP6C	5537	0.00212766	0.021276596	0.1
ANK2	287	TTN	7273	0.003012048	0.02892562	0.007207207
ANK3	288	ACTN2	88	0.043280182	0.049095607	0.00212766
ANK3	288	ANK1	286	0.005694761	0.07751938	0.242553191
ANK3	288	ANK2	287	0.004555809	0.028423773	0.063829787
ANK3	288	ATXN1	6310	0.054669704	0.031007752	0.008510638
ANK3	288	BCLAF1	9774	0.009111617	0.015503876	0.036170213

ANK3	288	BRAF	673	0.033029613	0.031007752	0.05106383
ANK3	288	CASR	846	0.01594533	0.018087855	0.065957447
ANK3	288	CDKN2A	1029	0.029612756	0.028423773	0.017021277
ANK3	288	CNTNAP2	26047	0.002277904	0.036175711	0.006382979
ANK3	288	COL7A1	1294	0.001138952	0.002583979	0.00212766
ANK3	288	FLT1	2321	0.007972665	0.046511628	0.006382979
ANK3	288	GRIN2A	2903	0.059225513	0.093023256	0.19787234
ANK3	288	GRM8	2918	0.003416856	0.007751938	0.055319149
ANK3	288	HDAC9	9734	0.007972665	0.11627907	0.059574468
ANK3	288	KDR	3791	0.043280182	0.025839793	0.038297872
ANK3	288	LIFR	3977	0.001138952	0.005167959	0.006382979
ANK3	288	MDC1	9656	0.039863326	0.012919897	0.063829787
ANK3	288	NF1	4763	0.027334852	0.043927649	0.121276596
ANK3	288	NID1	4811	0.001138952	0.002583979	0.006382979
ANK3	288	NOS1	4842	0.012528474	0.041343669	0.057446809
ANK3	288	NOTCH4	4855	0.004555809	0.007751938	0.025531915
ANK3	288	NRAS	4893	0.005694761	0.062015504	0.085106383
ANK3	288	PLCB1	23236	0.004555809	0.005167959	0.059574468
ANK3	288	PTEN	5728	0.014806378	0.056847545	0.108510638
ANK3	288	PTPRB	5787	0.003416856	0.005167959	0.012765957
ANK3	288	TP53	7157	0.001138952	0.010335917	0.006382979
ANK3	288	TRPV5	56302	0.001138952	0.002583979	0.00212766
ANK3	288	TTN	7273	0.001138952	0.028423773	0.00212766
ANK3	288	ZFHX3	463	0.019362187	0.03875969	0.010638298
APOB	338	ACTN2	88	0.001390821	0.005555556	0.002403846
APOB	338	ANK1	286	0.001390821	0.027777778	0.007211538

APOB	338	BCLAF1	9774	0.001390821	0.011111111	0.007211538
APOB	338	BRAF	673	0.006954103	0.005555556	0.012019231
APOB	338	CASR	846	0.002781641	0.033333333	0.028846154
APOB	338	CDKN2A	1029	0.008344924	0.005555556	0.012019231
APOB	338	DSCAM	1826	0.002781641	0.022222222	0.021634615
APOB	338	FLT1	2321	0.037552156	0.022222222	0.033653846
APOB	338	GRIN2A	2903	0.016689847	0.055555556	0.067307692
APOB	338	GRM7	2917	0.001390821	0.016666667	0.036057692
APOB	338	GRM8	2918	0.005563282	0.005555556	0.084134615
APOB	338	HDAC9	9734	0.008344924	0.094444444	0.024038462
APOB	338	KDR	3791	0.019471488	0.016666667	0.012019231
APOB	338	NF1	4763	0.001390821	0.027777778	0.016826923
APOB	338	NOS1	4842	0.002781641	0.038888889	0.009615385
APOB	338	PRLR	5618	0.001390821	0.011111111	0.002403846
APOB	338	PTEN	5728	0.012517385	0.05	0.052884615
APOB	338	SCN5A	6331	0.006954103	0.016666667	0.002403846
APOB	338	ZFHX3	463	0.005563282	0.027777778	0.012019231
BCLAF1	9774	BRAF	673	0.015360983	0.004444444	0.007633588
BCLAF1	9774	FLT1	2321	0.015360983	0.013333333	0.011450382
BCLAF1	9774	GRIN2A	2903	0.003072197	0.022222222	0.061068702
BCLAF1	9774	HDAC9	9734	0.001536098	0.084444444	0.041984733
BCLAF1	9774	KDR	3791	0.003072197	0.013333333	0.064885496
BCLAF1	9774	MDC1	9656	0.003072197	0.008888889	0.007633588
BCLAF1	9774	NRAS	4893	0.25499232	0.102222222	0.053435115
BCLAF1	9774	PTEN	5728	0.004608295	0.026666667	0.019083969
BRAF	673	FLT1	2321	0.002197802	0.028469751	0.012269939

BRAF	673	GRIN2A	2903	0.001098901	0.060498221	0.04601227
BRAF	673	HDAC9	9734	0.003296703	0.071174377	0.009202454
BRAF	673	MDC1	9656	0.056043956	0.007117438	0.06595092
CASR	846	BRAF	673	0.004040404	0.016666667	0.107632094
CASR	846	FLT1	2321	0.006060606	0.011111111	0.031311155
CASR	846	GRIN2A	2903	0.022222222	0.027777778	0.031311155
CASR	846	KDR	3791	0.002020202	0.011111111	0.043052838
CASR	846	NF1	4763	0.002020202	0.022222222	0.011741683
CASR	846	PTEN	5728	0.002020202	0.044444444	0.02739726
CDKN2A	1029	ATXN1	6310	0.009259259	0.021052632	0.00660066
CDKN2A	1029	MDC1	9656	0.00154321	0.042105263	0.03630363
CFTR	1080	ACTN2	88	0.00968523	0.034136546	0.007058824
CFTR	1080	ANK2	287	0.002421308	0.008032129	0.004705882
CFTR	1080	APOB	338	0.001210654	0.050200803	0.004705882
CFTR	1080	BCLAF1	9774	0.012106538	0.018072289	0.056470588
CFTR	1080	BRAF	673	0.008474576	0.014056225	0.148235294
CFTR	1080	CASR	846	0.054479419	0.034136546	0.096470588
CFTR	1080	CDKN2A	1029	0.013317191	0.008032129	0.056470588
CFTR	1080	CNTNAP2	26047	0.002421308	0.036144578	0.004705882
CFTR	1080	CNTNAP4	85445	0.001210654	0.014056225	0.009411765
CFTR	1080	DSCAM	1826	0.006053269	0.040160643	0.009411765
CFTR	1080	FLT1	2321	0.015738499	0.014056225	0.044705882
CFTR	1080	GRIN2A	2903	0.12590799	0.104417671	0.388235294
CFTR	1080	GRM7	2917	0.001210654	0.006024096	0.028235294
CFTR	1080	GRM8	2918	0.002421308	0.010040161	0.061176471
CFTR	1080	HDAC9	9734	0.006053269	0.120481928	0.082352941

CFTR	1080	IL7R	3575	0.002421308	0.004016064	0.023529412
CFTR	1080	KDR	3791	0.006053269	0.010040161	0.014117647
CFTR	1080	MDC1	9656	0.02905569	0.014056225	0.018823529
CFTR	1080	NF1	4763	0.018159806	0.03815261	0.117647059
CFTR	1080	NOS1	4842	0.033898305	0.048192771	0.037647059
CFTR	1080	NOTCH4	4855	0.002421308	0.014056225	0.035294118
CFTR	1080	NRAS	4893	0.002421308	0.018072289	0.077647059
CFTR	1080	PAK7	57144	0.001210654	0.012048193	0.014117647
CFTR	1080	PLCB1	23236	0.001210654	0.006024096	0.049411765
CFTR	1080	PPP6C	5537	0.001210654	0.014056225	0.068235294
CFTR	1080	PRLR	5618	0.007263923	0.012048193	0.054117647
CFTR	1080	PTEN	5728	0.035108959	0.064257028	0.174117647
CFTR	1080	PTPRB	5787	0.002421308	0.012048193	0.035294118
CFTR	1080	SALL1	6299	0.004842615	0.010040161	0.002352941
CFTR	1080	TP53	7157	0.002421308	0.006024096	0.004705882
CFTR	1080	TRPV6	55503	0.003631961	0.002008032	0.002352941
CFTR	1080	ZFHX3	463	0.008474576	0.048192771	0.016470588
COL14A1	7373	ANK1	286	0.005486968	0.046511628	0.005797101
COL14A1	7373	CASR	846	0.001371742	0.03875969	0.092753623
COL14A1	7373	DSCAM	1826	0.002743484	0.007751938	0.037681159
COL14A1	7373	GRIN2A	2903	0.013717421	0.069767442	0.115942029
COL14A1	7373	HDAC9	9734	0.001371742	0.046511628	0.008695652
COL14A1	7373	MDC1	9656	0.001371742	0.023255814	0.037681159
COL14A1	7373	NF1	4763	0.001371742	0.03875969	0.043478261
COL14A1	7373	NOS1	4842	0.002743484	0.015503876	0.008695652
COL14A1	7373	PRLR	5618	0.001371742	0.015503876	0.008695652

COL14A1	7373	PTEN	5728	0.002743484	0.062015504	0.191304348
COL3A1	1281	ACTN2	88	0.012544803	0.03	0.003322259
COL3A1	1281	BRAF	673	0.069892473	0.01	0.119601329
COL3A1	1281	DSCAM	1826	0.02688172	0.08	0.03986711
COL3A1	1281	GRIN2A	2903	0.02688172	0.07	0.102990033
COL3A1	1281	MDC1	9656	0.007168459	0.02	0.016611296
COL3A1	1281	NF1	4763	0.005376344	0.08	0.205980066
COL3A1	1281	NOS1	4842	0.001792115	0.04	0.003322259
COL3A1	1281	PRLR	5618	0.003584229	0.03	0.006644518
COL3A1	1281	PTEN	5728	0.001792115	0.04	0.159468439
COL3A1	1281	TTN	7273	0.001792115	0.04	0.006644518
COL4A4	1286	COL4A5	1287	0.002747253	0.013157895	0.010869565
COL4A4	1286	HDAC9	9734	0.002747253	0.065789474	0.010869565
COL4A4	1286	MDC1	9656	0.002747253	0.013157895	0.010869565
COL4A4	1286	PTEN	5728	0.002747253	0.092105263	0.125
COL4A5	1287	ACTN2	88	0.001089325	0.019736842	0.002469136
COL4A5	1287	BCLAF1	9774	0.001089325	0.009868421	0.02962963
COL4A5	1287	BRAF	673	0.004357298	0.128289474	0.091358025
COL4A5	1287	CASR	846	0.003267974	0.042763158	0.130864198
COL4A5	1287	CDKN2A	1029	0.002178649	0.006578947	0.014814815
COL4A5	1287	DSCAM	1826	0.002178649	0.019736842	0.059259259
COL4A5	1287	FLT1	2321	0.004357298	0.016447368	0.014814815
COL4A5	1287	GRIN2A	2903	0.008714597	0.072368421	0.222222222
COL4A5	1287	GRM7	2917	0.002178649	0.009868421	0.032098765
COL4A5	1287	HDAC9	9734	0.001089325	0.115131579	0.041975309
COL4A5	1287	MDC1	9656	0.008714597	0.026315789	0.044444444

COL4A5	1287	NID1	4811	0.020697168	0.006578947	0.039506173
COL4A5	1287	NOS1	4842	0.003267974	0.055921053	0.034567901
COL4A5	1287	NRAS	4893	0.009803922	0.177631579	0.111111111
COL4A5	1287	PTEN	5728	0.001089325	0.072368421	0.2
COL4A5	1287	TTN	7273	0.001089325	0.039473684	0.007407407
COL4A5	1287	ZFHX3	463	0.002178649	0.023026316	0.007407407
COL7A1	1294	ANK1	286	0.001068376	0.02016129	0.008
COL7A1	1294	BCLAF1	9774	0.004273504	0.004032258	0.036
COL7A1	1294	BRAF	673	0.012820513	0.068548387	0.086
COL7A1	1294	CASR	846	0.001068376	0.02016129	0.09
COL7A1	1294	CDKN2A	1029	0.002136752	0.008064516	0.028
COL7A1	1294	COL4A1	1282	0.44017094	0.27016129	0.184
COL7A1	1294	DSCAM	1826	0.002136752	0.024193548	0.038
COL7A1	1294	FLT1	2321	0.002136752	0.02016129	0.022
COL7A1	1294	GRIN2A	2903	0.007478632	0.084677419	0.24
COL7A1	1294	GRM8	2918	0.004273504	0.004032258	0.056
COL7A1	1294	HDAC9	9734	0.003205128	0.084677419	0.068
COL7A1	1294	KDR	3791	0.002136752	0.008064516	0.018
COL7A1	1294	LIFR	3977	0.002136752	0.012096774	0.008
COL7A1	1294	MDC1	9656	0.004273504	0.016129032	0.026
COL7A1	1294	NF1	4763	0.001068376	0.060483871	0.098
COL7A1	1294	NID1	4811	0.152777778	0.060483871	0.046
COL7A1	1294	NOS1	4842	0.001068376	0.040322581	0.032
COL7A1	1294	NRAS	4893	0.007478632	0.072580645	0.07
COL7A1	1294	PTEN	5728	0.007478632	0.129032258	0.296
COL7A1	1294	ZFHX3	463	0.003205128	0.02016129	0.01

DSP	1832	ANK1	286	0.001126126	0.034852547	0.023668639
DSP	1832	BCLAF1	9774	0.006756757	0.008042895	0.044378698
DSP	1832	BRAF	673	0.023648649	0.034852547	0.044378698
DSP	1832	CASR	846	0.007882883	0.021447721	0.056213018
DSP	1832	CDKN2A	1029	0.004504505	0.010723861	0.017751479
DSP	1832	DSCAM	1826	0.003378378	0.016085791	0.014792899
DSP	1832	FLT1	2321	0.006756757	0.024128686	0.032544379
DSP	1832	GRIN2A	2903	0.031531532	0.077747989	0.171597633
DSP	1832	GRM8	2918	0.003378378	0.002680965	0.071005917
DSP	1832	HDAC9	9734	0.021396396	0.120643432	0.109467456
DSP	1832	KDR	3791	0.006756757	0.021447721	0.01183432
DSP	1832	MDC1	9656	0.025900901	0.016085791	0.088757396
DSP	1832	NF1	4763	0.006756757	0.034852547	0.136094675
DSP	1832	NOS1	4842	0.012387387	0.053619303	0.044378698
DSP	1832	NOTCH4	4855	0.003378378	0.002680965	0.014792899
DSP	1832	NRAS	4893	0.004504505	0.024128686	0.068047337
DSP	1832	PLCB1	23236	0.001126126	0.002680965	0.041420118
DSP	1832	PRLR	5618	0.001126126	0.010723861	0.047337278
DSP	1832	PTEN	5728	0.012387387	0.034852547	0.085798817
DSP	1832	PTPRB	5787	0.002252252	0.013404826	0.038461538
DSP	1832	TTN	7273	0.004504505	0.037533512	0.00295858
DSP	1832	ZFHX3	463	0.038288288	0.042895442	0.035502959
EPHB6	2051	ACTN2	88	0.036117381	0.037142857	0.030805687
EPHB6	2051	ANK1	286	0.001128668	0.048571429	0.045023697
EPHB6	2051	ANK2	287	0.001128668	0.028571429	0.011848341
EPHB6	2051	ATXN1	6310	0.0248307	0.02	0.002369668

EPHB6	2051	BCLAF1	9774	0.009029345	0.011428571	0.059241706
EPHB6	2051	BRAF	673	0.007900677	0.051428571	0.073459716
EPHB6	2051	CASR	846	0.009029345	0.025714286	0.165876777
EPHB6	2051	CDKN2A	1029	0.013544018	0.005714286	0.009478673
EPHB6	2051	CHD6	84181	0.001128668	0.005714286	0.007109005
EPHB6	2051	CNTNAP4	85445	0.001128668	0.005714286	0.014218009
EPHB6	2051	DSCAM	1826	0.001128668	0.025714286	0.004739336
EPHB6	2051	FLT1	2321	0.033860045	0.022857143	0.165876777
EPHB6	2051	GRIN2A	2903	0.031602709	0.102857143	0.225118483
EPHB6	2051	GRM7	2917	0.001128668	0.005714286	0.035545024
EPHB6	2051	GRM8	2918	0.003386005	0.002857143	0.056872038
EPHB6	2051	HDAC9	9734	0.011286682	0.165714286	0.078199052
EPHB6	2051	KDR	3791	0.005643341	0.02	0.045023697
EPHB6	2051	LIFR	3977	0.002257336	0.008571429	0.023696682
EPHB6	2051	MDC1	9656	0.039503386	0.028571429	0.063981043
EPHB6	2051	NF1	4763	0.001128668	0.051428571	0.120853081
EPHB6	2051	NID1	4811	0.003386005	0.002857143	0.002369668
EPHB6	2051	NOS1	4842	0.009029345	0.042857143	0.075829384
EPHB6	2051	NOTCH4	4855	0.001128668	0.008571429	0.016587678
EPHB6	2051	NRAS	4893	0.004514673	0.042857143	0.168246445
EPHB6	2051	PRLR	5618	0.001128668	0.034285714	0.018957346
EPHB6	2051	PTEN	5728	0.046275395	0.051428571	0.104265403
EPHB6	2051	PTPRB	5787	0.004514673	0.022857143	0.035545024
EPHB6	2051	SCN5A	6331	0.002257336	0.014285714	0.007109005
EPHB6	2051	TP53	7157	0.018058691	0.002857143	0.007109005
EPHB6	2051	TRPV5	56302	0.001128668	0.002857143	0.002369668

EPHB6	2051	TTN	7273	0.005643341	0.034285714	0.016587678
EPHB6	2051	ZFHX3	463	0.018058691	0.028571429	0.026066351
ERBB4	2066	ACTN2	88	0.100571429	0.044270833	0.006564551
ERBB4	2066	ANK1	286	0.004571429	0.049479167	0.002188184
ERBB4	2066	BCLAF1	9774	0.001142857	0.0078125	0.080962801
ERBB4	2066	BRAF	673	0.019428571	0.083333333	0.041575492
ERBB4	2066	CASR	846	0.011428571	0.020833333	0.094091904
ERBB4	2066	CDKN2A	1029	0.006857143	0.002604167	0.010940919
ERBB4	2066	CNTNAP2	26047	0.003428571	0.044270833	0.010940919
ERBB4	2066	COL14A1	7373	0.001142857	0.010416667	0.002188184
ERBB4	2066	COL4A1	1282	0.001142857	0.091145833	0.004376368
ERBB4	2066	DSCAM	1826	0.005714286	0.0390625	0.035010941
ERBB4	2066	FLT1	2321	0.086857143	0.049479167	0.089715536
ERBB4	2066	GRIN2A	2903	0.061714286	0.098958333	0.284463895
ERBB4	2066	GRM8	2918	0.001142857	0.005208333	0.043763676
ERBB4	2066	HDAC9	9734	0.002285714	0.127604167	0.035010941
ERBB4	2066	KDR	3791	0.017142857	0.026041667	0.026258206
ERBB4	2066	LIFR	3977	0.002285714	0.002604167	0.004376368
ERBB4	2066	NF1	4763	0.006857143	0.057291667	0.078774617
ERBB4	2066	NOS1	4842	0.006857143	0.041666667	0.059080963
ERBB4	2066	NRAS	4893	0.003428571	0.151041667	0.455142232
ERBB4	2066	OSMR	9180	0.001142857	0.0078125	0.032822757
ERBB4	2066	PAK7	57144	0.001142857	0.013020833	0.01750547
ERBB4	2066	PRLR	5618	0.002285714	0.0078125	0.048140044
ERBB4	2066	PTEN	5728	0.018285714	0.044270833	0.172866521
ERBB4	2066	PTPRB	5787	0.001142857	0.033854167	0.015317287

ERBB4	2066	TRPV5	56302	0.001142857	0.015625	0.002188184
ERBB4	2066	TTN	7273	0.002285714	0.046875	0.008752735
ERBB4	2066	ZFHX3	463	0.003428571	0.018229167	0.004376368
F13A1	2162	DSCAM	1826	0.003144654	0.033333333	0.030927835
F13A1	2162	FLT1	2321	0.001572327	0.016666667	0.013745704
F13A1	2162	HDAC9	9734	0.003144654	0.075	0.020618557
F13A1	2162	MDC1	9656	0.009433962	0.025	0.013745704
F13A1	2162	PTEN	5728	0.003144654	0.041666667	0.137457045
FLNB	2317	ACTN2	88	0.007853403	0.050505051	0.00802139
FLNB	2317	ANK1	286	0.005235602	0.037037037	0.013368984
FLNB	2317	ANK2	287	0.002617801	0.050505051	0.00802139
FLNB	2317	APOB	338	0.001308901	0.04040404	0.002673797
FLNB	2317	BCLAF1	9774	0.011780105	0.006734007	0.069518717
FLNB	2317	BRAF	673	0.009162304	0.023569024	0.034759358
FLNB	2317	CASR	846	0.015706806	0.02020202	0.114973262
FLNB	2317	DSCAM	1826	0.002617801	0.047138047	0.032085561
FLNB	2317	FLT1	2321	0.009162304	0.02020202	0.016042781
FLNB	2317	GRIN2A	2903	0.031413613	0.077441077	0.114973262
FLNB	2317	GRM7	2917	0.005235602	0.003367003	0.018716578
FLNB	2317	GRM8	2918	0.036649215	0.01010101	0.050802139
FLNB	2317	HDAC9	9734	0.003926702	0.101010101	0.021390374
FLNB	2317	MDC1	9656	0.017015707	0.013468013	0.053475936
FLNB	2317	NF1	4763	0.006544503	0.016835017	0.072192513
FLNB	2317	NOS1	4842	0.010471204	0.03030303	0.026737968
FLNB	2317	NOTCH4	4855	0.001308901	0.013468013	0.00802139
FLNB	2317	NRAS	4893	0.010471204	0.013468013	0.090909091

FLNB	2317	PRLR	5618	0.001308901	0.013468013	0.018716578
FLNB	2317	PTEN	5728	0.007853403	0.063973064	0.227272727
FLNB	2317	SCN5A	6331	0.002617801	0.01010101	0.002673797
FLNB	2317	SELE	6401	0.081151832	0.003367003	0.00802139
FLNB	2317	TTN	7273	0.054973822	0.050505051	0.00802139
FLNB	2317	ZFHX3	463	0.002617801	0.023569024	0.016042781
FLT1	2321	ACTN2	88	0.004801921	0.019157088	0.002673797
FLT1	2321	BRAF	673	0.007202881	0.049808429	0.045454545
FLT1	2321	CASR	846	0.00120048	0.007662835	0.032085561
FLT1	2321	CDKN2A	1029	0.024009604	0.003831418	0.005347594
FLT1	2321	DSCAM	1826	0.003601441	0.022988506	0.005347594
FLT1	2321	GRIN2A	2903	0.014405762	0.068965517	0.14171123
FLT1	2321	KDR	3791	0.008403361	0.011494253	0.010695187
FLT1	2321	LIFR	3977	0.00120048	0.007662835	0.005347594
FLT1	2321	MDC1	9656	0.022809124	0.011494253	0.021390374
FLT1	2321	NF1	4763	0.00240096	0.019157088	0.010695187
FLT1	2321	NOS1	4842	0.00240096	0.026819923	0.013368984
FLT1	2321	NOTCH4	4855	0.00120048	0.011494253	0.002673797
FLT1	2321	PRLR	5618	0.00120048	0.01532567	0.056149733
FLT1	2321	PTEN	5728	0.00120048	0.045977011	0.122994652
GRIN2A	2903	ACTN2	88	0.056899004	0.035555556	0.002352941
GRIN2A	2903	ATXN1	6310	0.06685633	0.017777778	0.002352941
GRIN2A	2903	BCLAF1	9774	0.00284495	0.004444444	0.025882353
GRIN2A	2903	BRAF	673	0.022759602	0.084444444	0.089411765
GRIN2A	2903	CASR	846	0.011379801	0.026666667	0.143529412
GRIN2A	2903	CDKN2A	1029	0.001422475	0.022222222	0.018823529

GRIN2A	2903	DSCAM	1826	0.001422475	0.031111111	0.025882353
GRIN2A	2903	FLT1	2321	0.036984353	0.04	0.028235294
GRIN2A	2903	GRM7	2917	0.001422475	0.008888889	0.035294118
GRIN2A	2903	GRM8	2918	0.001422475	0.008888889	0.030588235
GRIN2A	2903	IL7R	3575	0.00284495	0.004444444	0.004705882
GRIN2A	2903	KDR	3791	0.038406828	0.031111111	0.023529412
GRIN2A	2903	MDC1	9656	0.018492176	0.035555556	0.054117647
GRIN2A	2903	NF1	4763	0.004267425	0.057777778	0.167058824
GRIN2A	2903	NOS1	4842	0.0056899	0.048888889	0.018823529
GRIN2A	2903	NRAS	4893	0.004267425	0.057777778	0.084705882
GRIN2A	2903	PLCB1	23236	0.001422475	0.004444444	0.016470588
GRIN2A	2903	PTEN	5728	0.019914651	0.031111111	0.101176471
GRIN2A	2903	SELE	6401	0.009957326	0.004444444	0.002352941
GRIN2A	2903	TTN	7273	0.011379801	0.04	0.011764706
GRIN2A	2903	ZFHX3	463	0.0056899	0.026666667	0.002352941
GRM3	2913	ACTN2	88	0.013513514	0.031007752	0.017241379
GRM3	2913	ANK1	286	0.002252252	0.046511628	0.00862069
GRM3	2913	BRAF	673	0.011261261	0.007751938	0.034482759
GRM3	2913	CASR	846	0.006756757	0.007751938	0.051724138
GRM3	2913	CNTNAP2	26047	0.006756757	0.015503876	0.00862069
GRM3	2913	DSCAM	1826	0.002252252	0.007751938	0.060344828
GRM3	2913	FLT1	2321	0.015765766	0.031007752	0.051724138
GRM3	2913	GRIN2A	2903	0.022522523	0.139534884	0.284482759
GRM3	2913	GRM7	2917	0.002252252	0.015503876	0.163793103
GRM3	2913	GRM8	2918	0.004504505	0.100775194	0.387931034
GRM3	2913	HDAC9	9734	0.006756757	0.093023256	0.025862069

GRM3	2913	KDR	3791	0.006756757	0.015503876	0.051724138
GRM3	2913	NF1	4763	0.006756757	0.069767442	0.077586207
GRM3	2913	NOS1	4842	0.009009009	0.015503876	0.017241379
GRM3	2913	NRAS	4893	0.002252252	0.007751938	0.034482759
GRM3	2913	PTEN	5728	0.011261261	0.069767442	0.068965517
GRM7	2917	ANK1	286	0.002232143	0.01910828	0.003003003
GRM7	2917	BRAF	673	0.002232143	0.012738854	0.069069069
GRM7	2917	CASR	846	0.004464286	0.01910828	0.018018018
GRM7	2917	CDKN2A	1029	0.020089286	0.012738854	0.003003003
GRM7	2917	FLT1	2321	0.024553571	0.012738854	0.015015015
GRM7	2917	GRIN2A	2903	0.024553571	0.089171975	0.618618619
GRM7	2917	GRM8	2918	0.006696429	0.025477707	0.09009009
GRM7	2917	KCNQ5	56479	0.0625	0.006369427	0.003003003
GRM7	2917	KDR	3791	0.015625	0.012738854	0.009009009
GRM7	2917	MDC1	9656	0.020089286	0.012738854	0.048048048
GRM7	2917	NRAS	4893	0.002232143	0.063694268	0.273273273
GRM7	2917	PTEN	5728	0.004464286	0.038216561	0.123123123
GRM7	2917	TTN	7273	0.042410714	0.012738854	0.009009009
GRM7	2917	ZFHX3	463	0.004464286	0.012738854	0.006006006
HDAC9	9734	BRAF	673	0.004830918	0.018987342	0.07253886
HDAC9	9734	DSCAM	1826	0.001610306	0.025316456	0.002590674
HDAC9	9734	KDR	3791	0.006441224	0.012658228	0.020725389
HDAC9	9734	MDC1	9656	0.011272142	0.018987342	0.028497409
HDAC9	9734	NRAS	4893	0.001610306	0.170886076	0.03626943
HDAC9	9734	PTEN	5728	0.004830918	0.03164557	0.007772021
IL7R	3575	ATXN1	6310	0.015332198	0.024390244	0.002590674

IL7R	3575	CDKN2A	1029	0.008517888	0.008130081	0.002590674
IL7R	3575	FLT1	2321	0.071550256	0.056910569	0.051813472
IL7R	3575	GRIN2A	2903	0.059625213	0.130081301	0.347150259
IL7R	3575	KDR	3791	0.045996593	0.032520325	0.033678756
IL7R	3575	MDC1	9656	0.039182283	0.040650407	0.051813472
IL7R	3575	NF1	4763	0.010221465	0.016260163	0.064766839
IL7R	3575	NOS1	4842	0.001703578	0.040650407	0.031088083
KALRN	8997	ACTN2	88	0.006369427	0.06440678	0.00528169
KALRN	8997	ANK1	286	0.001273885	0.033898305	0.003521127
KALRN	8997	ANK2	287	0.002547771	0.030508475	0.003521127
KALRN	8997	ATXN1	6310	0.007643312	0.023728814	0.001760563
KALRN	8997	BCLAF1	9774	0.005095541	0.006779661	0.01584507
KALRN	8997	BRAF	673	0.003821656	0.030508475	0.024647887
KALRN	8997	CASR	846	0.001273885	0.013559322	0.01584507
KALRN	8997	CDKN2A	1029	0.011464968	0.013559322	0.003521127
KALRN	8997	CNTNAP2	26047	0.001273885	0.030508475	0.008802817
KALRN	8997	DSCAM	1826	0.001273885	0.044067797	0.049295775
KALRN	8997	FLT1	2321	0.010191083	0.027118644	0.008802817
KALRN	8997	GRIN2A	2903	0.008917197	0.074576271	0.088028169
KALRN	8997	GRM8	2918	0.003821656	0.006779661	0.017605634
KALRN	8997	HDAC9	9734	0.005095541	0.088135593	0.026408451
KALRN	8997	KDR	3791	0.01910828	0.020338983	0.01056338
KALRN	8997	MDC1	9656	0.008917197	0.020338983	0.033450704
KALRN	8997	NF1	4763	0.001273885	0.06440678	0.01584507
KALRN	8997	NRAS	4893	0.005095541	0.050847458	0.063380282
KALRN	8997	PLCB1	23236	0.001273885	0.006779661	0.01584507

KALRN	8997	PTEN	5728	0.007643312	0.057627119	0.059859155
KALRN	8997	SCN5A	6331	0.001273885	0.003389831	0.001760563
KALRN	8997	TTN	7273	0.001273885	0.027118644	0.003521127
KCNQ3	3786	ANK1	286	0.00304878	0.02027027	0.007407407
KCNQ3	3786	ATXN1	6310	0.006097561	0.02027027	0.007407407
KCNQ3	3786	BRAF	673	0.009146341	0.033783784	0.111111111
KCNQ3	3786	DSCAM	1826	0.00304878	0.013513514	0.014814815
KCNQ3	3786	FLT1	2321	0.00304878	0.013513514	0.02962963
KCNQ3	3786	GRIN2A	2903	0.015243902	0.040540541	0.074074074
KCNQ3	3786	HDAC9	9734	0.00304878	0.033783784	0.014814815
KCNQ3	3786	KCNQ5	56479	0.487804878	0.371621622	0.755555556
KCNQ3	3786	KDR	3791	0.00304878	0.013513514	0.037037037
KCNQ3	3786	NF1	4763	0.006097561	0.013513514	0.022222222
KCNQ3	3786	NOS1	4842	0.00304878	0.060810811	0.022222222
KDR	3791	BCLAF1	9774	0.001666667	0.005681818	0.018050542
KDR	3791	BRAF	673	0.011666667	0.073863636	0.014440433
KDR	3791	CASR	846	0.001666667	0.017045455	0.039711191
KDR	3791	FLT1	2321	0.008333333	0.005681818	0.010830325
KDR	3791	GRIN2A	2903	0.016666667	0.147727273	0.093862816
KDR	3791	GRM7	2917	0.001666667	0.005681818	0.007220217
KDR	3791	GRM8	2918	0.001666667	0.005681818	0.007220217
KDR	3791	MDC1	9656	0.026666667	0.045454545	0.036101083
KDR	3791	NF1	4763	0.001666667	0.011363636	0.003610108
KDR	3791	NOS1	4842	0.001666667	0.017045455	0.010830325
KDR	3791	NRAS	4893	0.001666667	0.051136364	0.064981949
KDR	3791	PRLR	5618	0.003333333	0.011363636	0.028880866

KDR	3791	PTEN	5728	0.001666667	0.034090909	0.115523466
KSR2	283455	ACTN2	88	0.033248082	0.035087719	0.003766478
KSR2	283455	ATXN1	6310	0.037084399	0.020467836	0.003766478
KSR2	283455	BCLAF1	9774	0.023017903	0.00877193	0.043314501
KSR2	283455	BRAF	673	0.126598465	0.070175439	0.472693032
KSR2	283455	CASR	846	0.003836317	0.014619883	0.04519774
KSR2	283455	CDKN2A	1029	0.006393862	0.005847953	0.035781544
KSR2	283455	DSCAM	1826	0.001278772	0.035087719	0.015065913
KSR2	283455	FLT1	2321	0.046035806	0.032163743	0.06779661
KSR2	283455	GRIN2A	2903	0.042199488	0.076023392	0.150659134
KSR2	283455	GRM8	2918	0.001278772	0.00877193	0.039548023
KSR2	283455	HDAC9	9734	0.017902813	0.125730994	0.128060264
KSR2	283455	KDR	3791	0.012787724	0.01754386	0.028248588
KSR2	283455	LIFR	3977	0.020460358	0.002923977	0.007532957
KSR2	283455	MDC1	9656	0.035805627	0.040935673	0.071563089
KSR2	283455	NF1	4763	0.010230179	0.011695906	0.04519774
KSR2	283455	NOS1	4842	0.001278772	0.052631579	0.015065913
KSR2	283455	NOTCH4	4855	0.001278772	0.002923977	0.005649718
KSR2	283455	NRAS	4893	0.023017903	0.067251462	0.320150659
KSR2	283455	PRLR	5618	0.001278772	0.01754386	0.035781544
KSR2	283455	PTEN	5728	0.008951407	0.046783626	0.11299435
KSR2	283455	TTN	7273	0.001278772	0.00877193	0.003766478
KSR2	283455	ZFHX3	463	0.006393862	0.026315789	0.015065913
LAMA2	3908	ACTN2	88	0.008588957	0.06504065	0.002123142
LAMA2	3908	ATXN1	6310	0.013496933	0.008130081	0.002123142
LAMA2	3908	BCLAF1	9774	0.002453988	0.012195122	0.014861996

LAMA2	3908	BRAF	673	0.007361963	0.044715447	0.142250531
LAMA2	3908	CASR	846	0.007361963	0.024390244	0.171974522
LAMA2	3908	DSCAM	1826	0.004907975	0.052845528	0.140127389
LAMA2	3908	FLT1	2321	0.026993865	0.008130081	0.014861996
LAMA2	3908	GRIN2A	2903	0.014723926	0.073170732	0.129511677
LAMA2	3908	HDAC9	9734	0.004907975	0.077235772	0.038216561
LAMA2	3908	MDC1	9656	0.008588957	0.016260163	0.076433121
LAMA2	3908	NF1	4763	0.004907975	0.040650407	0.055201699
LAMA2	3908	NID1	4811	0.06993865	0.004065041	0.012738854
LAMA2	3908	NOS1	4842	0.001226994	0.052845528	0.016985138
LAMA2	3908	PRLR	5618	0.002453988	0.004065041	0.031847134
LAMA2	3908	PTEN	5728	0.002453988	0.044715447	0.112526539
LAMA2	3908	ZFHX3	463	0.001226994	0.036585366	0.006369427
LIFR	3977	ACTN2	88	0.027642276	0.044025157	0.005905512
LIFR	3977	ANK2	287	0.001626016	0.012578616	0.007874016
LIFR	3977	BCLAF1	9774	0.011382114	0.003144654	0.059055118
LIFR	3977	BRAF	673	0.019512195	0.009433962	0.031496063
LIFR	3977	CASR	846	0.01300813	0.022012579	0.066929134
LIFR	3977	COL4A5	1287	0.01300813	0.003144654	0.001968504
LIFR	3977	DSCAM	1826	0.003252033	0.081761006	0.035433071
LIFR	3977	FLT1	2321	0.048780488	0.018867925	0.047244094
LIFR	3977	GRIN2A	2903	0.048780488	0.091194969	0.188976378
LIFR	3977	HDAC9	9734	0.001626016	0.072327044	0.059055118
LIFR	3977	KDR	3791	0.017886179	0.009433962	0.019685039
LIFR	3977	MDC1	9656	0.053658537	0.009433962	0.082677165
LIFR	3977	NF1	4763	0.004878049	0.018867925	0.045275591

LIFR	3977	NOS1	4842	0.024390244	0.050314465	0.041338583
LIFR	3977	NRAS	4893	0.001626016	0.037735849	0.147637795
LIFR	3977	OSMR	9180	0.003252033	0.022012579	0.037401575
LIFR	3977	PRLR	5618	0.003252033	0.01572327	0.05511811
LIFR	3977	PTEN	5728	0.011382114	0.044025157	0.135826772
LIFR	3977	PTPRB	5787	0.001626016	0.006289308	0.021653543
LIFR	3977	SELE	6401	0.071544715	0.009433962	0.013779528
LIFR	3977	SNCAIP	9627	0.003252033	0.006289308	0.057086614
LIFR	3977	TTN	7273	0.001626016	0.050314465	0.005905512
LIFR	3977	ZFHX3	463	0.003252033	0.056603774	0.00984252
MACF1	23499	ACTN2	88	0.003787879	0.034632035	0.211764706
MACF1	23499	BRAF	673	0.002525253	0.03030303	0.020588235
MACF1	23499	CNTNAP2	26047	0.01010101	0.108225108	0.002941176
MACF1	23499	CNTNAP4	85445	0.001262626	0.051948052	0.008823529
MACF1	23499	COL4A1	1282	0.001262626	0.004329004	0.058823529
MACF1	23499	COL4A5	1287	0.001262626	0.012987013	0.023529412
MACF1	23499	FLT1	2321	0.001262626	0.012987013	0.020588235
MACF1	23499	GRIN2A	2903	0.003787879	0.082251082	0.091176471
MACF1	23499	HDAC9	9734	0.001262626	0.082251082	0.076470588
MACF1	23499	KDR	3791	0.001262626	0.012987013	0.011764706
MACF1	23499	MDC1	9656	0.011363636	0.021645022	0.032352941
MACF1	23499	NRAS	4893	0.001262626	0.021645022	0.023529412
MACF1	23499	PTEN	5728	0.008838384	0.047619048	0.126470588
MACF1	23499	PTPRB	5787	0.001262626	0.004329004	0.002941176
MYO3A	53904	ACTN2	88	0.011299435	0.041958042	0.003159558
MYO3A	53904	ANK1	286	0.002259887	0.024475524	0.009478673

MYO3A	53904	BCLAF1	9774	0.001129944	0.01048951	0.006319115
MYO3A	53904	BRAF	673	0.002259887	0.024475524	0.042654028
MYO3A	53904	CASR	846	0.002259887	0.013986014	0.04107425
MYO3A	53904	CDKN2A	1029	0.028248588	0.006993007	0.034755134
MYO3A	53904	COL14A1	7373	0.002259887	0.017482517	0.001579779
MYO3A	53904	FLT1	2321	0.029378531	0.038461538	0.012638231
MYO3A	53904	GRIN2A	2903	0.021468927	0.055944056	0.173775671
MYO3A	53904	HDAC9	9734	0.002259887	0.08041958	0.086887836
MYO3A	53904	IL7R	3575	0.001129944	0.003496503	0.001579779
MYO3A	53904	KDR	3791	0.023728814	0.034965035	0.039494471
MYO3A	53904	MDC1	9656	0.013559322	0.045454545	0.012638231
MYO3A	53904	NF1	4763	0.005649718	0.017482517	0.048973144
MYO3A	53904	NID1	4811	0.001129944	0.003496503	0.001579779
MYO3A	53904	NOS1	4842	0.002259887	0.052447552	0.023696682
MYO3A	53904	NRAS	4893	0.010169492	0.045454545	0.033175355
MYO3A	53904	PPP6C	5537	0.005649718	0.006993007	0.045813586
MYO3A	53904	PTEN	5728	0.006779661	0.027972028	0.093206951
MYO3A	53904	PTPRB	5787	0.001129944	0.003496503	0.006319115
MYO3A	53904	TTN	7273	0.004519774	0.055944056	0.001579779
MYO3A	53904	ZFHX3	463	0.003389831	0.017482517	0.004739336
NEBL	10529	ACTN2	88	0.039906103	0.046511628	0.119318182
NEBL	10529	ANK1	286	0.002347418	0.015503876	0.011363636
NEBL	10529	ANK2	287	0.002347418	0.015503876	0.008522727
NEBL	10529	ATXN1	6310	0.436619718	0.289405685	0.002840909
NEBL	10529	BCLAF1	9774	0.008215962	0.015503876	0.053977273
NEBL	10529	BRAF	673	0.03286385	0.018087855	0.048295455

NEBL	10529	CASR	846	0.008215962	0.028423773	0.136363636
NEBL	10529	CDKN2A	1029	0.022300469	0.007751938	0.036931818
NEBL	10529	CNTNAP2	26047	0.001173709	0.059431525	0.002840909
NEBL	10529	CNTNAP4	85445	0.001173709	0.010335917	0.002840909
NEBL	10529	DSCAM	1826	0.001173709	0.023255814	0.036931818
NEBL	10529	FLT1	2321	0.023474178	0.023255814	0.022727273
NEBL	10529	GRIN2A	2903	0.072769953	0.098191214	0.224431818
NEBL	10529	GRM7	2917	0.001173709	0.002583979	0.042613636
NEBL	10529	GRM8	2918	0.003521127	0.007751938	0.085227273
NEBL	10529	HDAC9	9734	0.004694836	0.142118863	0.088068182
NEBL	10529	KALRN	8997	0.001173709	0.005167959	0.036931818
NEBL	10529	KDR	3791	0.008215962	0.002583979	0.011363636
NEBL	10529	LIFR	3977	0.001173709	0.005167959	0.014204545
NEBL	10529	MDC1	9656	0.039906103	0.018087855	0.068181818
NEBL	10529	NF1	4763	0.014084507	0.059431525	0.136363636
NEBL	10529	NID1	4811	0.003521127	0.03875969	0.014204545
NEBL	10529	NOS1	4842	0.018779343	0.043927649	0.051136364
NEBL	10529	NOTCH4	4855	0.001173709	0.007751938	0.011363636
NEBL	10529	NRAS	4893	0.023474178	0.028423773	0.116477273
NEBL	10529	PLCB1	23236	0.003521127	0.005167959	0.036931818
NEBL	10529	PRLR	5618	0.008215962	0.023255814	0.113636364
NEBL	10529	PTEN	5728	0.021126761	0.031007752	0.119318182
NEBL	10529	PTPRB	5787	0.004694836	0.005167959	0.005681818
NEBL	10529	SELE	6401	0.008215962	0.002583979	0.002840909
NEBL	10529	SNCAIP	9627	0.001173709	0.005167959	0.059659091
NEBL	10529	TTN	7273	0.005868545	0.049095607	0.068181818

NEBL	10529	ZFHX3	463	0.012910798	0.036175711	0.017045455
NF1	4763	FLT1	2321	0.008810573	0.032894737	0.01
NF1	4763	KDR	3791	0.004405286	0.006578947	0.0125
NOS1	4842	ACTN2	88	0.049531459	0.024691358	0.013452915
NOS1	4842	ATXN1	6310	0.026773762	0.027777778	0.002242152
NOS1	4842	BCLAF1	9774	0.017402945	0.00617284	0.042600897
NOS1	4842	BRAF	673	0.084337349	0.037037037	0.053811659
NOS1	4842	CASR	846	0.026773762	0.033950617	0.076233184
NOS1	4842	CDKN2A	1029	0.004016064	0.012345679	0.022421525
NOS1	4842	FLT1	2321	0.021419009	0.015432099	0.02690583
NOS1	4842	GRIN2A	2903	0.044176707	0.132716049	0.091928251
NOS1	4842	GRM8	2918	0.001338688	0.00617284	0.049327354
NOS1	4842	HDAC9	9734	0.00669344	0.086419753	0.033632287
NOS1	4842	KDR	3791	0.032128514	0.030864198	0.029147982
NOS1	4842	LIFR	3977	0.001338688	0.00308642	0.015695067
NOS1	4842	MDC1	9656	0.038821954	0.015432099	0.109865471
NOS1	4842	NF1	4763	0.009370817	0.049382716	0.069506726
NOS1	4842	NID1	4811	0.001338688	0.00308642	0.002242152
NOS1	4842	NOTCH4	4855	0.002677376	0.00617284	0.01793722
NOS1	4842	NRAS	4893	0.016064257	0.024691358	0.627802691
NOS1	4842	PRLR	5618	0.001338688	0.018518519	0.044843049
NOS1	4842	PTEN	5728	0.008032129	0.043209877	0.179372197
NOS1	4842	SCN5A	6331	0.009370817	0.012345679	0.076233184
NOS1	4842	SELE	6401	0.013386881	0.00308642	0.00896861
NOS1	4842	SNCAIP	9627	0.001338688	0.00308642	0.047085202
NOS1	4842	TTN	7273	0.009370817	0.058641975	0.080717489

NOS1	4842	ZFHX3	463	0.005354752	0.040123457	0.006726457
NOTCH4	4855	ANK1	286	0.00482509	0.042918455	0.014736842
NOTCH4	4855	BCLAF1	9774	0.001206273	0.017167382	0.008421053
NOTCH4	4855	BRAF	673	0.00482509	0.008583691	0.004210526
NOTCH4	4855	DSCAM	1826	0.007237636	0.021459227	0.008421053
NOTCH4	4855	FLT1	2321	0.026537998	0.021459227	0.008421053
NOTCH4	4855	GRIN2A	2903	0.002412545	0.081545064	0.073684211
NOTCH4	4855	HDAC9	9734	0.001206273	0.103004292	0.008421053
NOTCH4	4855	KDR	3791	0.003618818	0.008583691	0.008421053
NOTCH4	4855	MDC1	9656	0.010856454	0.021459227	0.056842105
NOTCH4	4855	NF1	4763	0.003618818	0.008583691	0.044210526
NOTCH4	4855	NRAS	4893	0.1013269	0.025751073	0.021052632
NOTCH4	4855	PTEN	5728	0.006031363	0.042918455	0.021052632
NOTCH4	4855	TRPV5	56302	0.001206273	0.004291845	0.002105263
NOTCH4	4855	TTN	7273	0.001206273	0.042918455	0.002105263
NRAS	4893	BCLAF1	9774	0.012485812	0.005494505	0.007507508
NRAS	4893	DSCAM	1826	0.002270148	0.008241758	0.031531532
NRAS	4893	GRIN2A	2903	0.001135074	0.002747253	0.003003003
NRAS	4893	MDC1	9656	0.026106697	0.010989011	0.073573574
NRAS	4893	PTEN	5728	0.001135074	0.065934066	0.093093093
OSMR	9180	BRAF	673	0.010771993	0.020134228	0.007832898
OSMR	9180	CDKN2A	1029	0.003590664	0.026845638	0.033942559
OSMR	9180	DSCAM	1826	0.003590664	0.020134228	0.031331593
OSMR	9180	FLT1	2321	0.019748654	0.020134228	0.01305483
OSMR	9180	GRIN2A	2903	0.016157989	0.087248322	0.075718016
OSMR	9180	KDR	3791	0.008976661	0.013422819	0.036553525

OSMR	9180	MDC1	9656	0.017953321	0.033557047	0.148825065
OSMR	9180	NF1	4763	0.001795332	0.020134228	0.020887728
OSMR	9180	NOS1	4842	0.007181329	0.020134228	0.020887728
OSMR	9180	NOTCH4	4855	0.001795332	0.013422819	0.002610966
OSMR	9180	NRAS	4893	0.001795332	0.013422819	0.208877285
OSMR	9180	PRLR	5618	0.001795332	0.006711409	0.015665796
PAK7	57144	ACTN2	88	0.008951407	0.042372881	0.16254417
PAK7	57144	ATXN1	6310	0.023017903	0.016949153	0.001766784
PAK7	57144	BCLAF1	9774	0.003836317	0.008474576	0.03180212
PAK7	57144	BRAF	673	0.023017903	0.033898305	0.058303887
PAK7	57144	CASR	846	0.006393862	0.025423729	0.040636042
PAK7	57144	CDKN2A	1029	0.003836317	0.008474576	0.007067138
PAK7	57144	CNTNAP2	26047	0.001278772	0.055084746	0.001766784
PAK7	57144	FLT1	2321	0.007672634	0.033898305	0.019434629
PAK7	57144	GRIN2A	2903	0.020460358	0.050847458	0.121908127
PAK7	57144	HDAC9	9734	0.006393862	0.118644068	0.033568905
PAK7	57144	KDR	3791	0.002557545	0.016949153	0.022968198
PAK7	57144	MDC1	9656	0.024296675	0.012711864	0.042402827
PAK7	57144	NF1	4763	0.00511509	0.012711864	0.026501767
PAK7	57144	NOS1	4842	0.003836317	0.046610169	0.028268551
PAK7	57144	NRAS	4893	0.001278772	0.021186441	0.14664311
PAK7	57144	PRLR	5618	0.002557545	0.008474576	0.014134276
PAK7	57144	PTEN	5728	0.007672634	0.021186441	0.106007067
PAK7	57144	SCN5A	6331	0.002557545	0.004237288	0.030035336
PAK7	57144	TTN	7273	0.002557545	0.050847458	0.051236749
PAK7	57144	ZFHX3	463	0.007672634	0.008474576	0.001766784

PLCB1	23236	ANK1	286	0.001818182	0.015625	0.002873563
PLCB1	23236	BRAF	673	0.058181818	0.03125	0.057471264
PLCB1	23236	CASR	846	0.014545455	0.03125	0.097701149
PLCB1	23236	CDKN2A	1029	0.003636364	0.010416667	0.022988506
PLCB1	23236	DSCAM	1826	0.010909091	0.010416667	0.048850575
PLCB1	23236	FLT1	2321	0.034545455	0.026041667	0.00862069
PLCB1	23236	GRIN2A	2903	0.025454545	0.083333333	0.117816092
PLCB1	23236	GRM7	2917	0.003636364	0.015625	0.034482759
PLCB1	23236	HDAC9	9734	0.012727273	0.104166667	0.060344828
PLCB1	23236	KDR	3791	0.001818182	0.005208333	0.011494253
PLCB1	23236	MDC1	9656	0.016363636	0.036458333	0.043103448
PLCB1	23236	NOS1	4842	0.003636364	0.041666667	0.022988506
PLCB1	23236	NRAS	4893	0.005454545	0.046875	0.094827586
PLCB1	23236	PTEN	5728	0.001818182	0.026041667	0.143678161
PLCB1	23236	TTN	7273	0.001818182	0.052083333	0.005747126
PPP6C	5537	ATXN1	6310	0.012820513	0.005617978	0.003676471
PPP6C	5537	BRAF	673	0.006410256	0.016853933	0.036764706
PPP6C	5537	DSCAM	1826	0.003205128	0.039325843	0.014705882
PPP6C	5537	GRIN2A	2903	0.003205128	0.04494382	0.099264706
PPP6C	5537	HDAC9	9734	0.001602564	0.101123596	0.088235294
PPP6C	5537	MDC1	9656	0.014423077	0.011235955	0.102941176
PPP6C	5537	NF1	4763	0.001602564	0.005617978	0.025735294
PPP6C	5537	NRAS	4893	0.017628205	0.028089888	0.025735294
PRLR	5618	ACTN2	88	0.028058361	0.038194444	0.013856813
PRLR	5618	ANK1	286	0.004489338	0.038194444	0.006928406
PRLR	5618	BCLAF1	9774	0.008978676	0.006944444	0.027713626

PRLR	5618	BRAF	673	0.046015713	0.010416667	0.115473441
PRLR	5618	CASR	846	0.012345679	0.020833333	0.060046189
PRLR	5618	CDKN2A	1029	0.012345679	0.006944444	0.006928406
PRLR	5618	CNTNAP2	26047	0.008978676	0.038194444	0.002309469
PRLR	5618	DSCAM	1826	0.001122334	0.045138889	0.032332564
PRLR	5618	FLT1	2321	0.03030303	0.013888889	0.043879908
PRLR	5618	GRIN2A	2903	0.02020202	0.086805556	0.173210162
PRLR	5618	GRM7	2917	0.002244669	0.010416667	0.025404157
PRLR	5618	GRM8	2918	0.003367003	0.041666667	0.048498845
PRLR	5618	HDAC9	9734	0.003367003	0.097222222	0.03926097
PRLR	5618	KDR	3791	0.01010101	0.010416667	0.025404157
PRLR	5618	MDC1	9656	0.042648709	0.024305556	0.060046189
PRLR	5618	NF1	4763	0.008978676	0.059027778	0.027713626
PRLR	5618	NOS1	4842	0.006734007	0.045138889	0.048498845
PRLR	5618	NOTCH4	4855	0.001122334	0.003472222	0.009237875
PRLR	5618	NRAS	4893	0.008978676	0.055555556	0.17551963
PRLR	5618	OSMR	9180	0.001122334	0.003472222	0.018475751
PRLR	5618	PTEN	5728	0.007856341	0.034722222	0.168591224
PRLR	5618	SCN5A	6331	0.003367003	0.024305556	0.002309469
PRLR	5618	SELE	6401	0.019079686	0.003472222	0.002309469
PRLR	5618	SNCAIP	9627	0.002244669	0.003472222	0.036951501
PRLR	5618	TTN	7273	0.003367003	0.052083333	0.009237875
PTEN	5728	ACTN2	88	0.019834711	0.013953488	0.005865103
PTEN	5728	BCLAF1	9774	0.00661157	0.004651163	0.011730205
PTEN	5728	BRAF	673	0.038016529	0.013953488	0.049853372
PTEN	5728	CDKN2A	1029	0.003305785	0.065116279	0.005865103

PTEN	5728	DSCAM	1826	0.001652893	0.004651163	0.04398827
PTEN	5728	FLT1	2321	0.008264463	0.009302326	0.026392962
PTEN	5728	GRIN2A	2903	0.011570248	0.055813953	0.102639296
PTEN	5728	KDR	3791	0.001652893	0.018604651	0.014662757
PTEN	5728	MDC1	9656	0.034710744	0.009302326	0.038123167
PTEN	5728	NF1	4763	0.003305785	0.013953488	0.023460411
PTEN	5728	NOS1	4842	0.003305785	0.023255814	0.038123167
PTEN	5728	NOTCH4	4855	0.001652893	0.013953488	0.002932551
PTEN	5728	NRAS	4893	0.008264463	0.093023256	0.090909091
PTEN	5728	PTPRB	5787	0.001652893	0.013953488	0.014662757
PTPRB	5787	ACTN2	88	0.003169572	0.020942408	0.00310559
PTPRB	5787	ANK2	287	0.001584786	0.031413613	0.049689441
PTPRB	5787	BRAF	673	0.028526149	0.036649215	0.086956522
PTPRB	5787	CDKN2A	1029	0.00792393	0.005235602	0.01242236
PTPRB	5787	DSCAM	1826	0.003169572	0.047120419	0.055900621
PTPRB	5787	FLT1	2321	0.011093502	0.005235602	0.00931677
PTPRB	5787	GRIN2A	2903	0.038034865	0.078534031	0.068322981
PTPRB	5787	KDR	3791	0.004754358	0.005235602	0.01242236
PTPRB	5787	LIFR	3977	0.003169572	0.005235602	0.00931677
PTPRB	5787	MDC1	9656	0.00792393	0.005235602	0.01552795
PTPRB	5787	NOS1	4842	0.004754358	0.031413613	0.02173913
PTPRB	5787	NRAS	4893	0.004754358	0.057591623	0.316770186
PTPRB	5787	PRLR	5618	0.001584786	0.015706806	0.065217391
PTPRB	5787	PTEN	5728	0.003169572	0.052356021	0.090062112
PTPRB	5787	SNCAIP	9627	0.001584786	0.010471204	0.01863354
PTPRB	5787	TTN	7273	0.001584786	0.041884817	0.00310559

SCN5A	6331	ACTN2	88	0.0125	0.027559055	0.010339734
SCN5A	6331	BCLAF1	9774	0.006818182	0.007874016	0.020679468
SCN5A	6331	BRAF	673	0.044318182	0.047244094	0.132939439
SCN5A	6331	CASR	846	0.010227273	0.007874016	0.026587888
SCN5A	6331	CDKN2A	1029	0.007954545	0.003937008	0.004431315
SCN5A	6331	CNTNAP2	26047	0.002272727	0.05511811	0.007385524
SCN5A	6331	GRIN2A	2903	0.0375	0.098425197	0.073855244
SCN5A	6331	HDAC9	9734	0.002272727	0.078740157	0.033973412
SCN5A	6331	KDR	3791	0.015909091	0.011811024	0.019202363
SCN5A	6331	MDC1	9656	0.015909091	0.031496063	0.038404727
SCN5A	6331	NF1	4763	0.001136364	0.019685039	0.033973412
SCN5A	6331	NOS1	4842	0.004545455	0.039370079	0.082717873
SCN5A	6331	NRAS	4893	0.002272727	0.039370079	0.094534712
SCN5A	6331	PLCB1	23236	0.003409091	0.007874016	0.023633678
SCN5A	6331	PTEN	5728	0.004545455	0.094488189	0.093057607
SCN5A	6331	SNCAIP	9627	0.001136364	0.007874016	0.036927622
SCN5A	6331	TTN	7273	0.0125	0.031496063	0.026587888
SCN5A	6331	ZFHX3	463	0.005681818	0.007874016	0.007385524
SNCAIP	9627	ACTN2	88	0.021546261	0.017123288	0.001818182
SNCAIP	9627	ATXN1	6310	0.031685678	0.020547945	0.007272727
SNCAIP	9627	BCLAF1	9774	0.012674271	0.006849315	0.021818182
SNCAIP	9627	BRAF	673	0.022813688	0.020547945	0.094545455
SNCAIP	9627	CASR	846	0.001267427	0.017123288	0.078181818
SNCAIP	9627	CDKN2A	1029	0.029150824	0.010273973	0.038181818
SNCAIP	9627	CNTNAP2	26047	0.001267427	0.034246575	0.001818182
SNCAIP	9627	DSCAM	1826	0.006337136	0.010273973	0.003636364

SNCAIP	9627	FLT1	2321	0.036755387	0.006849315	0.023636364
SNCAIP	9627	GRIN2A	2903	0.060836502	0.082191781	0.132727273
SNCAIP	9627	GRM7	2917	0.001267427	0.003424658	0.016363636
SNCAIP	9627	HDAC9	9734	0.006337136	0.140410959	0.105454545
SNCAIP	9627	KDR	3791	0.012674271	0.020547945	0.027272727
SNCAIP	9627	MDC1	9656	0.041825095	0.017123288	0.090909091
SNCAIP	9627	NF1	4763	0.003802281	0.023972603	0.045454545
SNCAIP	9627	NOS1	4842	0.003802281	0.030821918	0.029090909
SNCAIP	9627	NRAS	4893	0.006337136	0.058219178	0.014545455
SNCAIP	9627	PLCB1	23236	0.001267427	0.003424658	0.025454545
SNCAIP	9627	PRLR	5618	0.002534854	0.010273973	0.005454545
SNCAIP	9627	PTEN	5728	0.01774398	0.047945205	0.063636364
SNCAIP	9627	SCN5A	6331	0.006337136	0.010273973	0.018181818
SNCAIP	9627	TTN	7273	0.003802281	0.01369863	0.001818182
SNCAIP	9627	ZFHX3	463	0.006337136	0.020547945	0.007272727
TNR	7143	BRAF	673	0.027777778	0.023809524	0.058823529
TNR	7143	GRIN2A	2903	0.00308642	0.071428571	0.161764706
TNR	7143	MDC1	9656	0.015432099	0.03968254	0.007352941
TNR	7143	NOS1	4842	0.009259259	0.063492063	0.007352941
TNR	7143	NRAS	4893	0.00308642	0.007936508	0.036764706
TNR	7143	PTEN	5728	0.00308642	0.031746032	0.125
TP53	7157	BRAF	673	0.005194805	0.015625	0.006578947
TP53	7157	CASR	846	0.002597403	0.0234375	0.065789474
TP53	7157	CDKN2A	1029	0.005194805	0.0390625	0.118421053
TP53	7157	GRIN2A	2903	0.020779221	0.0703125	0.092105263
TP53	7157	KDR	3791	0.005194805	0.0234375	0.046052632

TP53	7157	MDC1	9656	0.293506494	0.21875	0.401315789
TP53	7157	NOS1	4842	0.002597403	0.03125	0.006578947
TP53	7157	NRAS	4893	0.01038961	0.0078125	0.013157895
TP53	7157	PTEN	5728	0.028571429	0.0390625	0.032894737
TRPV6	55503	ACTN2	88	0.01532567	0.042207792	0.008528785
TRPV6	55503	ANK1	286	0.002554278	0.029220779	0.010660981
TRPV6	55503	ATXN1	6310	0.03192848	0.012987013	0.004264392
TRPV6	55503	BCLAF1	9774	0.053639847	0.003246753	0.036247335
TRPV6	55503	BRAF	673	0.03192848	0.061688312	0.153518124
TRPV6	55503	CASR	846	0.057471264	0.038961039	0.300639659
TRPV6	55503	CDKN2A	1029	0.005108557	0.00974026	0.017057569
TRPV6	55503	CNTNAP2	26047	0.002554278	0.029220779	0.004264392
TRPV6	55503	COL14A1	7373	0.001277139	0.025974026	0.002132196
TRPV6	55503	DSCAM	1826	0.001277139	0.025974026	0.017057569
TRPV6	55503	FLT1	2321	0.045977011	0.029220779	0.068230277
TRPV6	55503	GRIN2A	2903	0.060025543	0.103896104	0.132196162
TRPV6	55503	GRM7	2917	0.002554278	0.006493506	0.051172708
TRPV6	55503	GRM8	2918	0.003831418	0.003246753	0.076759062
TRPV6	55503	HDAC9	9734	0.005108557	0.094155844	0.061833689
TRPV6	55503	KDR	3791	0.011494253	0.016233766	0.025586354
TRPV6	55503	LIFR	3977	0.001277139	0.003246753	0.010660981
TRPV6	55503	MDC1	9656	0.025542784	0.012987013	0.057569296
TRPV6	55503	NF1	4763	0.002554278	0.042207792	0.034115139
TRPV6	55503	NID1	4811	0.001277139	0.003246753	0.006396588
TRPV6	55503	NOS1	4842	0.01660281	0.029220779	0.044776119
TRPV6	55503	NOTCH4	4855	0.002554278	0.00974026	0.008528785

TRPV6	55503	NRAS	4893	0.01532567	0.071428571	0.125799574
TRPV6	55503	PAK7	57144	0.001277139	0.006493506	0.017057569
TRPV6	55503	PLCB1	23236	0.006385696	0.003246753	0.025586354
TRPV6	55503	PRLR	5618	0.003831418	0.006493506	0.042643923
TRPV6	55503	PTEN	5728	0.017879949	0.074675325	0.149253731
TRPV6	55503	PTPRB	5787	0.001277139	0.025974026	0.025586354
TRPV6	55503	SCN5A	6331	0.006385696	0.00974026	0.002132196
TRPV6	55503	TTN	7273	0.025542784	0.071428571	0.006396588
TRPV6	55503	ZFHX3	463	0.002554278	0.029220779	0.012793177
TTN	7273	ACTN2	88	0.31865285	0.071428571	0.00990099
TTN	7273	BRAF	673	0.018134715	0.015306122	0.01320132
TTN	7273	CASR	846	0.002590674	0.025510204	0.00660066
TTN	7273	DSCAM	1826	0.002590674	0.015306122	0.00330033
TTN	7273	FLT1	2321	0.002590674	0.020408163	0.00330033
TTN	7273	GRIN2A	2903	0.002590674	0.030612245	0.0330033
TTN	7273	HDAC9	9734	0.007772021	0.086734694	0.00330033
TTN	7273	KCNQ5	56479	0.051813472	0.005102041	0.00330033
TTN	7273	KDR	3791	0.010362694	0.025510204	0.00990099
TTN	7273	MDC1	9656	0.007772021	0.035714286	0.04950495
TTN	7273	PLCB1	23236	0.002590674	0.005102041	0.00330033
TTN	7273	PTEN	5728	0.007772021	0.040816327	0.00990099
